# Individual and joint exposure to air pollutants and patterns of multiple chronic conditions

**DOI:** 10.1038/s41598-024-73485-7

**Published:** 2024-09-30

**Authors:** Weifang Dai, Weina Xu, Jiayu Zhou, Shanna Liu, Qingli Zhou

**Affiliations:** 1 Department of Information Technology, The Fourth Affiliated Hospital of School of Medicine, and International School of Medicine, International Institutes of Medicine, Zhejiang University, Yiwu, 322000 China; 2 Department of Geriatric, Center for Regeneration and Aging Medicine，the Fourth Affiliated Hospital of School of Medicine, and International School of Medicine, International Institutes of Medicine, Zhejiang University, Yiwu, 322000 China; 3 School of Medicine, Shihezi University, Shihezi, Xinjiang, 832000 China

**Keywords:** Air pollutants, Multiple chronic conditions (MCC), Joint exposure, Aging population, National study, Environmental impact, Natural hazards, Diseases, Medical research, Risk factors

## Abstract

Existing research on the detrimental effects of air pollution and its mixture on multiple chronic conditions (MCC) is not yet fully recognized. Our objective was to examine if individual and joint exposure to air pollution is associated with the incidence and patterns of MCC. Totally 10,231 CHARLS 2015 participants aged over 45 years and 1,938 without MCC were followed up in 2018 and 2020. Residential-levelcumulative personal exposure concentrations of PM_1_, PM_10_, PM_2.5_, CO, O_3_, NO_2_, SO_2_, NO_3_^−^, Cl^−^, NH_4_^+^, and SO_4_^2−^ at the residential level were determined utilizing a spatio-temporal random forest model with a spatial resolution of 0.1° × 0.1°. In the cross-sectional and longitudinal research, logistic regression, cox regression analysis, and quantile g-computation were utilized to estimate the single and joint effect with MCC and its patterns, respectively. Interaction analyses and stratified analyses were also performed. A correlation was observed between the prevalence of cardiovascular illnesses and the presence of all 11 major air pollutants. PM_2.5_, PM_10_, NH_4_^+^, NO_3_^−^, CO, and SO_4_^2−^ are associated with an increased frequency of respiratory disorders. An increase of PM_2.5_, PM_1_, PM_10_, NO_2_, and SO_2_ (a 10 µg/m^3^ rise), CO (a 0.1 mg/m3 rise), and PMCs (Cl^−^, NH_4_^+^, NO_3_^−^, and SO_4_^2−^) (a 1 µg/m^3^ rise) corresponded to the HRs (95% CI) for developing MCC of 1.194 (95% CI: 1.043, 1.367), 1.362 (95% CI: 1.073, 1.728), 1.115 (95% CI: 1.026, 1.212), 1.443 (95% CI: 1.151, 1.808), 3.175 (95% CI: 2.291, 4.401), 1.272 (95% CI: 1.149,1.410), 1.382 (95% CI: 1.011, 1.888), 1.107 (95% CI: 1.003, 1.222), 1.035 (95% CI: 0.984, 1.088), and 1.122 (95% CI: 1.086, 1.160), respectively. SO_2_ was the predominant contributor to the combined effect (HR: 2.083, 95% CI: 1.659–2.508). Gender, age, drinking, and health status could modify the effects of air pollutants on MCC patterns. Long-term exposure to air pollution is correlated to the incidence and patterns of MCC in middle-aged and elderly Chinese individuals. Preventive methods are essential to safeguarding those susceptible to MCC.

**Supplementary Information:**

The online version contains supplementary material available at 10.1038/s41598-024-73485-7.

## Introduction

Multiple Chronic Conditions (MCC) refer to the presence of two or more chronic illnesses in one person^1^. As populations age, MCC has emerged as a significant global public health concern due to its increasing prevalence^2^. MCC is associated with poor patient outcomes, impairment, higher mortality risk, increased healthcare utilization, and substantial medical costs, which pose significant burdens on both individuals and healthcare systems^3,4^. In a systematic review and meta-analysis, the prevalence of multimorbidity among Chinese adults was reported to be 42.4%. The co-occurrence of several chronic illnesses in one person is often attributed to shared etiological factors^5,6^. Therefore, a deeper understanding of the clustering patterns of MCC is crucial for enhancing disease prevention strategies and improving conventional clinical practice^7^.

Among the factors contributing to the onset of MCC, air pollution has garnered significant attention as a non-negligible environmental factor, causing between 4 and 9 million premature deaths annually^8^. The elderly, due to their diminished physiological functions, are particularly susceptible to the adverse effects of air pollution^9,10^. Epidemiological studies have demonstrated a clear link between air pollution and negative health outcomes in the elderly, including respiratory disorders^11^, cardiovascular diseases^12,13^, and cognitive impairment^14^. Despite extensive evidence connecting air pollution with individual chronic diseases, comprehensive research on the impact of air pollution on individuals with MCC remains limited. This gap hampers our understanding of the full spectrum of health consequences associated with air pollution and impedes the development of effective prevention and control strategies^15^. Emerging research suggests that the health impacts of air pollution may vary by gender, highlighting the need for more nuanced investigations^16,17^.

To our knowledge, few studies have examined the role of air pollution in MCC patterns^18^. For instance, Hu et al.^19^ identified three MCC patterns but limited their analysis to PM_2.5_, potentially overlooking the influence of other air pollutants. Similarly, Amy et al.^20^ utilized data from the UK Biobank to explore the relationship between PM_2.5_, NO_2_, and MCC patterns using Exploratory Factor Analysis (EFA). However, their cross-sectional study design hindered definitive causal conclusions, and the specific demographic characteristics of the UK Biobank cohort introduced selection bias that may limit the generalizability of their findings.

To address this research gap, we utilized data from the China Health and Retirement Longitudinal Study (CHARLS), a nationally representative survey of middle-aged and older adults, to investigate the impact of 11 significant air pollutants and their mixtures on chronic disease markers. Latent class analysis (LCA) was employed in this study due to its effectiveness in identifying high-risk groups of older adults with multiple diseases, providing insights into the negative impact of specific disease combinations on elderly mortality, and categorizing MCC^21,22^. By integrating both cross-sectional and longitudinal study methods, we systematically assessed the complex relationship between single and combined air pollution exposure and multi-disease co-existing patterns. Our findings aim to provide robust scientific support and policy recommendations for improving air quality and reducing chronic disease burden in China.

## Methods

### Study population

The data for this investigation were derived from the China Health and Retirement Longitudinal Study (CHARLS), a nationally representative, prospective cohort study designed to collect comprehensive data on individuals aged 45 and older for geriatric research purposes. The baseline survey, conducted nationwide from 2011 to 2012, encompassed approximately 10,000 households and 450 villages/neighborhood committees across 150 regions, ensuring a broad representation of the Chinese middle-aged and elderly population. Participants were enrolled in the baseline survey from June 2011 to March 2012. Subsequent waves of the study, with follow-up evaluations, were conducted every two years, generating a rich dataset spanning the years 2013, 2015, 2018, and 2020.

**Fig. 1:**
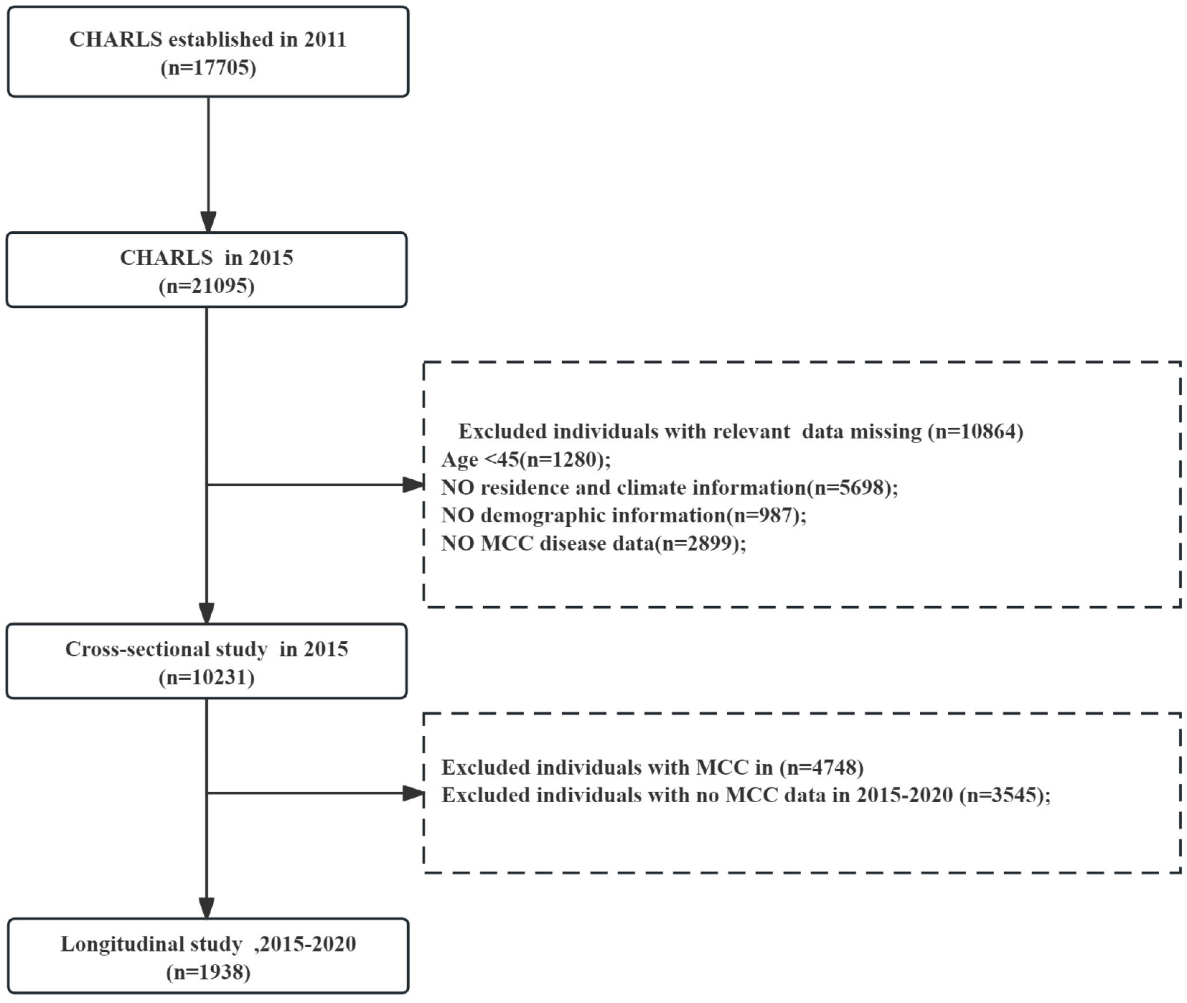
Flow chart.

In the current study, we extracted the data of new respondents during three waves (2015, 2018, and 2020) in the CHARLS after excluding participants who were under the age of 45, lacking essential information on morbidity, demographic factors, lifestyle details, and chronic diseases, as well as a limited number of individuals from regions where reliable air pollution exposure data were not available. This rigorous exclusion process aimed to ensure the validity and reliability of our findings. Ultimately, our study included 10,231 participants in the cross-sectional analysis and 1,938 participants in the longitudinal analysis. The participant selection procedure is illustrated in Fig. 1, which has been included in the supplementary material for clarity and transparency. The CHARLS 2015 dataset was used instead of the baseline to capture more recent population characteristics and better account for the complexity of population structure and individual variability, thus providing more accurate analysis results^23,24^.

The CHARLS project was approved by the Biomedical Ethics Review Committee at Peking University (Nu: IRB00001052-11015), and all participants provided written informed consent for the use of their data in research.

### The definition of MCC

MCC is the simultaneous presence of two or more chronic diseases, as outlined by the WHO and other epidemiological research^25^. The same definition of MCC was used in the current study. All participants in the survey were asked to confirm if they received a medical diagnosis for the following 14 chronic conditions: diabetes, hypertension, liver disease, heart disease, kidney failure, chronic pulmonary disease, cancer, dyslipidemia, digestive system illness, stroke, mental illnesses, memory-related disorders, arthritis, and asthma. If the participant responded with a ‘yes,’ this was accepted as the presence of chronic disease in that participant. All 14 chronic diseases were considered. Structured questionnaires were used in face-to-face interviews to collect self-reported symptoms of non-communicable illnesses. Individuals with cognitive impairment were not included in this process.

### Assessment of exposure to air pollution

The spatiotemporal random forest model was utilized to evaluate the concentrations of different pollutants at a geographical resolution of 0.1° × 0.1° using geocoded addresses. The model was created by combining ground monitoring air pollution data with satellite-derived optical depths of aerosols and other spatiotemporal predictors like cities, forests, and meteorological data. Next, the daily pollutant concentrations were estimated to determine the personal cumulative exposure of each participant over a 3-year period. Given privacy concerns regarding individual residential addresses, we resorted to city-level air pollution data as a proxy for individual-level exposure. Participants were matched to the average air pollution concentration at the city level. The air pollution exposure level for each individual was calculated as the average of the air pollution concentrations during the three years prior to either the occurrence of the outcome (disease) or the end of the cohort period (2020) if the individual did not develop the disease during the follow-up. Additionally, for subjects without diseases who were lost to follow-up, the exposure was averaged over the three years preceding their last follow-up visit from the time they entered the cohort. This ‘average’ represents the arithmetic mean of the air pollution data within the specified timeframes, serving as an estimate of an individual’s average exposure to air pollution during those periods.

The model achieved 10-fold cross-validation R2 values of 92% (10.76 µg/m^3^), 83% (9.50 µg/m^3^), 90% (21.12 µg/m^3^), 84% (10.07 µg/m^3^), 84% (7.99 µg/m^3^), 89% (15.77 µg/m^3^), 80% (0.29 mg/m^3^), 66% (2.3 µg/m^3^), 71% (4.3 µg/m^3^), 75% (6.6 µg/m^3^), and 74% (6.0 µg/m^3^) (Table S1). Further detailed information on the air pollution estimates is available in previous reports^26–33^.

### Latent class analysis (LCA) for four patterns

**Fig. 2:**
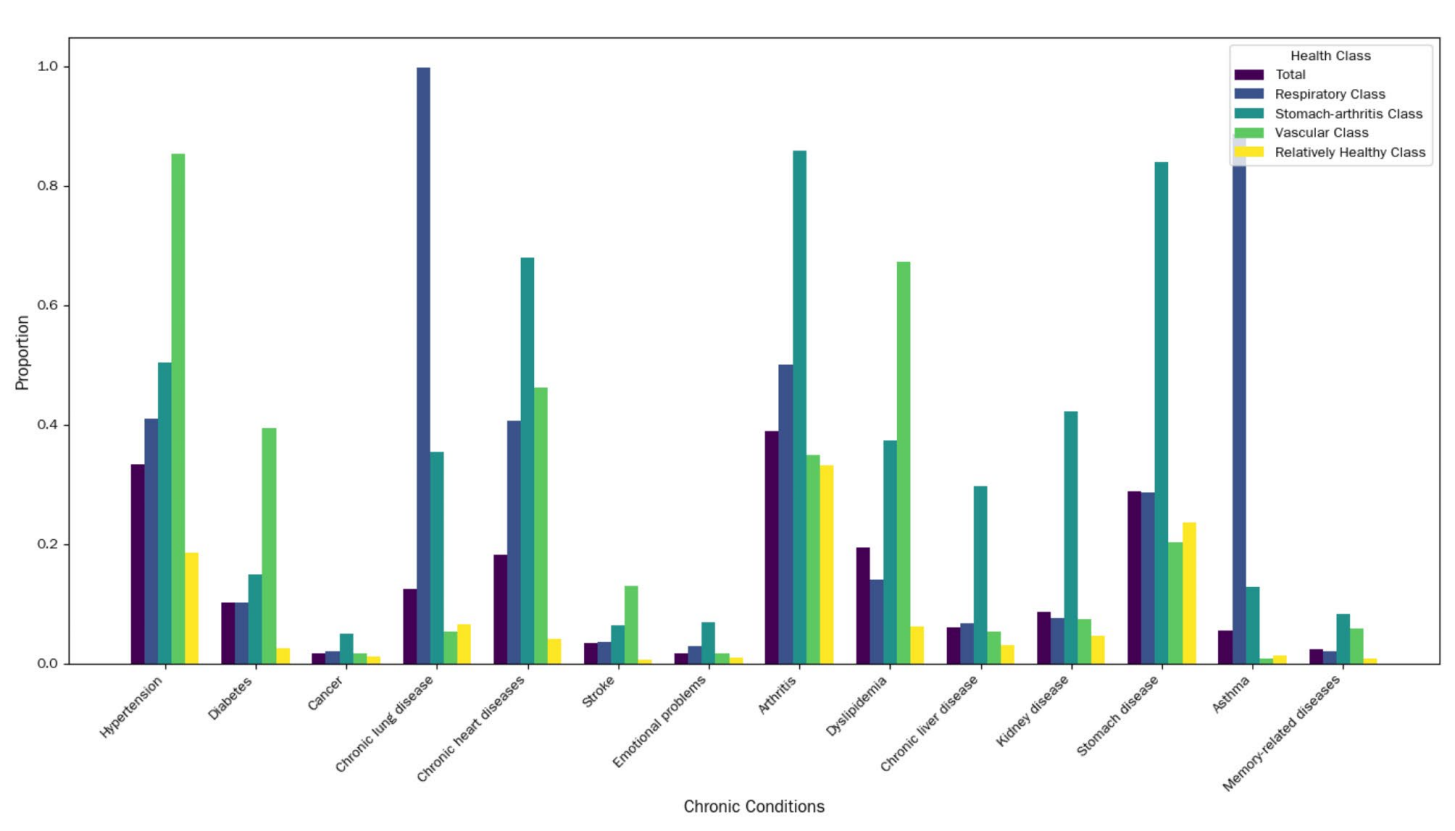
The four-class of MCC patterns.

LCA is a valuable technique for identifying unobserved subgroups within multivariate categorical data. In this study, we utilized LCA to categorize participants into distinct classes based on their profiles across 14 chronic diseases. The choice of four categories as the best-fitting model was guided by a comprehensive evaluation of several statistical criteria, including the Akaike Information Criterion (AIC), Bayesian Information Criterion (BIC), and entropy values, which indicated that the four-class model provided the optimal balance between model fit and interpretability^34^. Participants were assigned to different classes based on posterior membership probabilities, calculated using a model-based maximum likelihood procedure. The four identified classes are: The Relative Health class, encompassing 70.3% of participants, includes individuals with a lower risk of the 14 chronic diseases studied. The Cardiovascular Disease class, representing 16.7% of participants, includes those with conditions such as hypertension, diabetes, and heart attack. The Respiratory and Pulmonary Disease class, which includes 3.8% of participants, covers individuals with chronic lung disease and asthma. Lastly, the Stomach-Arthritis Disease class, comprising 9.1% of participants, includes individuals with gastric diseases and arthritis (Fig. 2). In addition, over half of the participants (51.1%) had two or more chronic conditions. The selection of these four categories was further validated by their clinical relevance and alignment with existing literature on MCC patterns^22,35^. The categories provide a meaningful framework for understanding the distribution and co-occurrence of chronic conditions, which can guide targeted interventions and inform clinical practice. Specific details of the screening process are in Table S2-3.

### Covariance

Various possible confounding variables were taken into account in the current investigation. The climatic parameters were obtained from the China Meteorological Administration’s website. Sociodemographic parameters considered in the study were age, gender, long-term residency (urban or rural), economic status (categorized into four economic zones: eastern, northeastern, central, and western zones), education level, marital status, and health insurance. The health behavior and lifestyle characteristics included were BMI, drinking and smoking habits, physical activity level, cooking fuel (solid and clean fuels), perceived health status (good, fair, and worse), depression (CESD-10 > 16:depressed symptoms^36^), and social activities (communication with friends, participation in community clubs, and volunteering). People who had ever smoked in any manner at any time in their lives were considered smokers. Participants’ drinking habits and types of drinking throughout the previous year were surveyed to ascertain their drinking status. To measure physical activity, the Physical Activity Questionnaire (IPAQ) was employed. The algorithm used to determine the Physical Activity (PA) score took several activity kinds into account. The time intervals utilized in the CHARLS were converted to midpoints, such as “≥ 4 hours” to 240 min, “≥ 2 hours to 180 minutes, and < 4 hours” to 180 min^37^.

###  Statistical analysis

First, baseline characteristics in the cross-sectional and longitudinal analytical samples were summarized according to MCC status and compared between individuals using the Student’s *t*-test, ANOVA, and Chi-square test, respectively. The correlations between different air contaminants were examined using Pearson correlation analysis.

In the cross-sectional study, Latent Class Analysis (LCA) was used to categorize various types of MCC based on the disease type and patient information. The analysis identified 14 distinct clustering patterns of chronic diseases among the participants, which were further broadly grouped into four categories. We utilized multivariate logistic regression to analyze the association between quartiles of average air pollutant concentration over the previous three years and the occurrence of these patterns. Two models were developed: a base model adjusted for seven variables (sex, education, age, BMI, marital status, air temperature, and relative humidity) and a main model that included additional variables (smoking, drinking, social activities, state of health, health insurance, place of residence, economic region, and physical activity).

For the longitudinal study, time-varying Cox regression models were employed to measure the time from the date of recruitment to the occurrence of MCC, death, or the last interview, whichever occurred first. The hazard ratio (HR) was calculated using the formula: h(t, x) = h0(t) exp (β_me_x_me_ + β_m_x_m_(t)), where h_0_(t) is the baseline hazard function and x_me_ denotes covariates that do not change over time, such as marital status, education level, sex, health status, BMI, lifestyle habits (including alcohol consumption and smoking), cooking fuel, place of residence, economic region category, public insurance, depression, physical activity, and social activity. The time-varying covariate β_m_x_m_(t) includes factors such as air pollution, temperature, relative humidity, and age^38^. The Cox proportional hazards regression model employed in this study did not violate the proportional hazards (PH) assumptions. After adjusting for confounders, air pollutant concentrations were treated as continuous variables and reported separately for pollutants such as PM_2.5_, PM_1_, PM_10_, NO_2_, and SO_2_ (each 10 µg/m^3^ increase), CO (each 0.1 mg/m^3^ increase), and PM_2.5_ chemical composition (PMCs, Cl^−^, NH_4_^+^, NO_3_^−^, and SO_4_^2−^) (each 1 µg/m^3^ increase) with results presented as Hazard Ratios (HR) and 95% confidence intervals (CI). Additionally, the nonlinear effects of air pollution concentrations were visualized using cubic splines.

Moreover, a quantile-based g-computation approach (R-package ‘qgcomp’) was utilized to analyze the combined effect of pollutant mixtures, which integrates weighted quantile sum (WQS) regression and g-computation to assess the impact of mixtures by one quantile on the outcomes^39–41^. In our study, four different mixtures were examined based on existing literature: one including all 11 pollution mixtures, another with particulate matter, 7 major air pollutants, and PMCs. Weights are assigned based on the overall composition of the most relevant results.

**Fig. 3:**
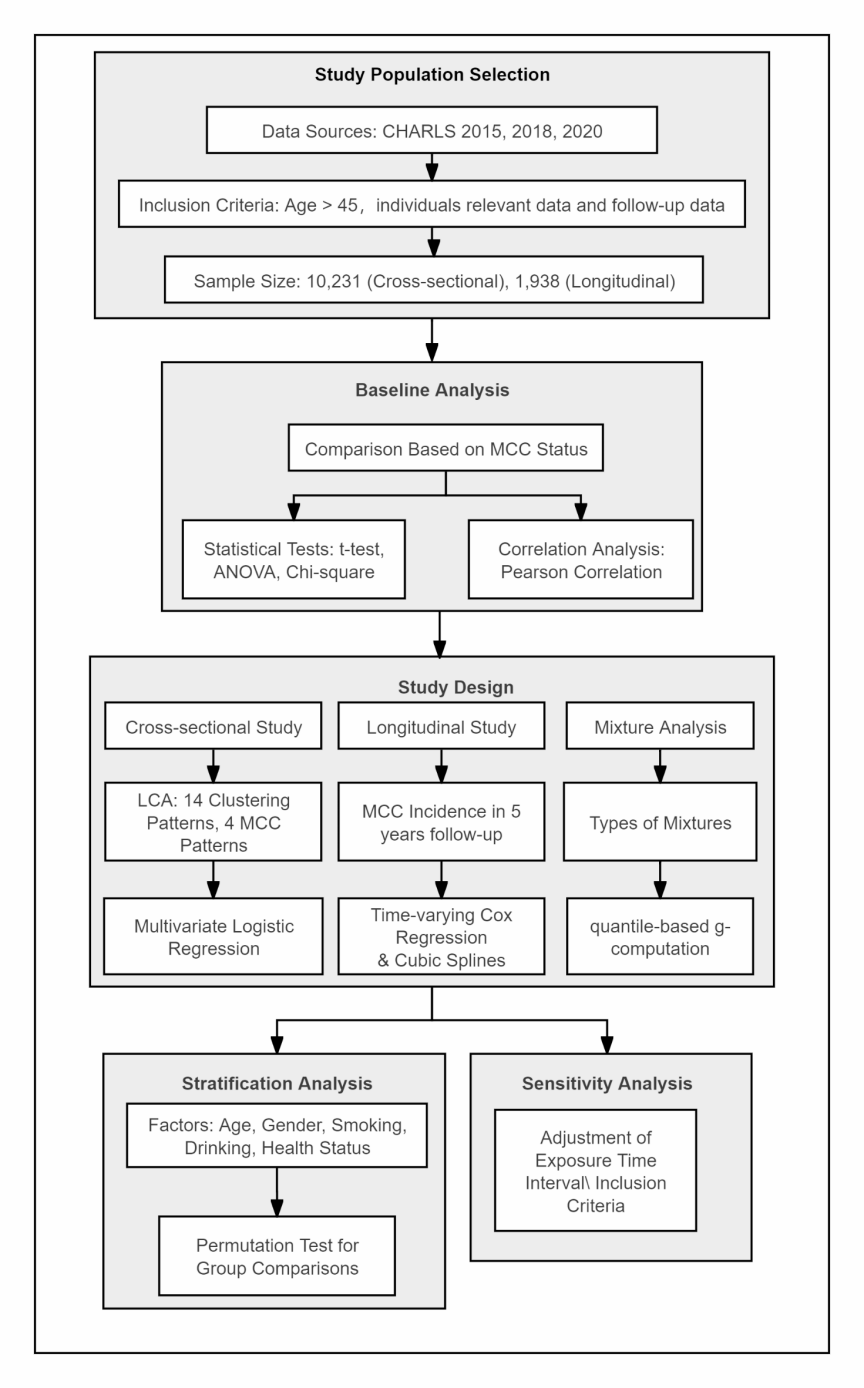
Research framework.

Multiple sensitivity analyses were conducted to assess the reliability and robustness of our results. First, the assessment of air pollution exposure concentration was adjusted from a three-year average to a two-year average to reinforce the validity of the conclusions. Next, participants with notably poor self-reported health or missing data were excluded to maintain the integrity of the dataset. Finally, a stratified analysis was performed to confirm the interaction effects observed in the study, with Fisher’s permutation test used to evaluate differences in the model coefficients by simulation^42^. The details of the research framework are presented in Fig. 3.

All methods were conducted in accordance with the relevant guidance and regulations. ALL studies were conducted using Mplus 7.8 and Stata 16.0. Geographic information system (GIS) analysis was conducted using ArcGIS Pro 2.1 to visualize the geospatial distribution. Results with a two-sided test and a P value below 0.05 were deemed statistically significant.

## Results

### Study population

**Fig. 4:**
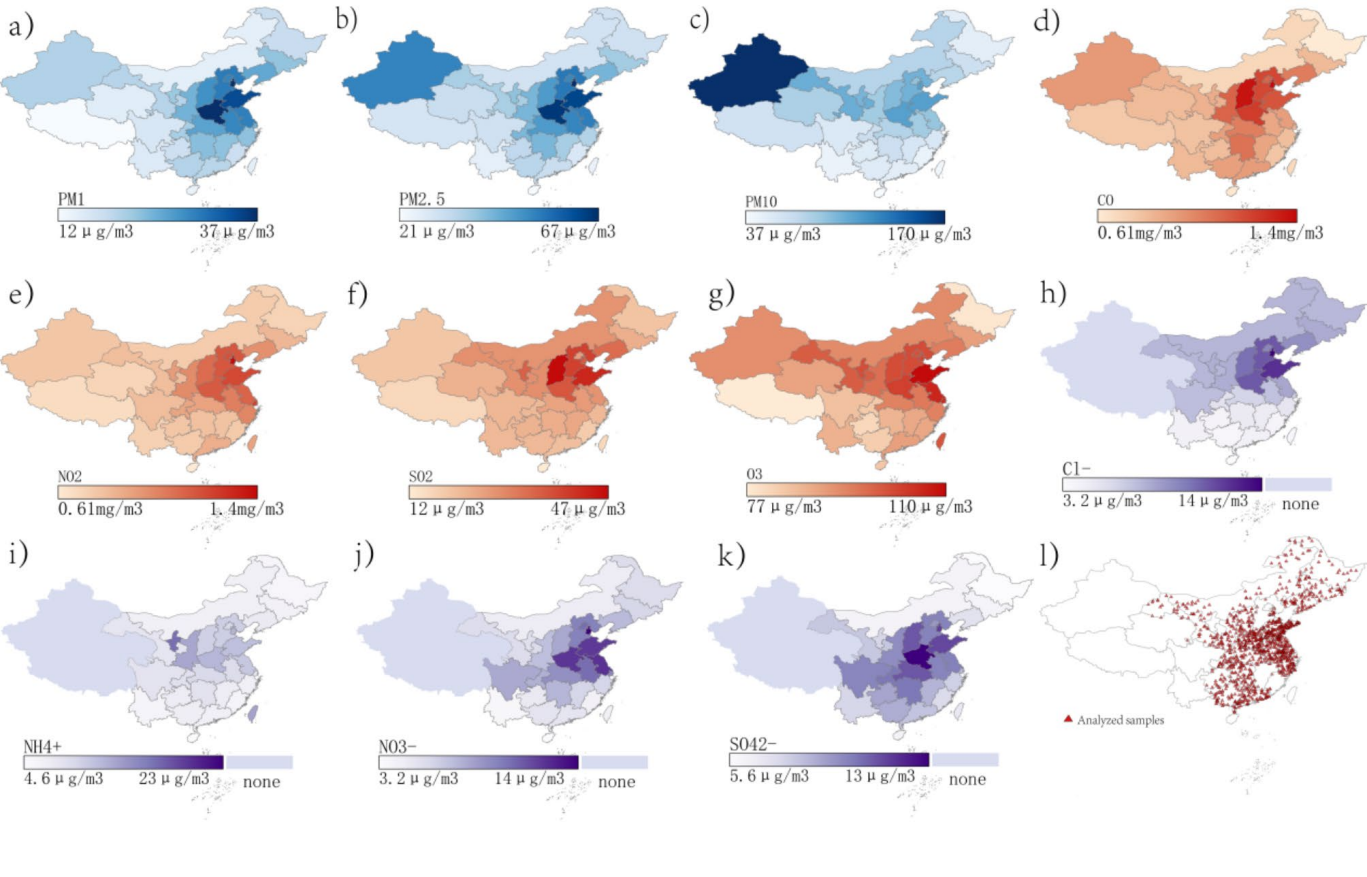
Geospatial distribution of analyzed samples and air pollution exposure. *Notes*: Data visualization analysis was used by ArcGIS Pro 2.1. Abbreviations: P25, P50, P75, Lower, middle and higher quartiles of variables; IQR, inter-quartile range; PM_2.5_, particle with aerodynamic diameter ≤ 2.5 μm; PM_1_, particle with aerodynamic diameter ≤ 1 μm; PM_10_, particle with aerodynamic diameter ≤ 10 μm; CO, carbonic oxide; O_3_, ozone; Cl^−^, chloride; NH_4_^+^, ammonium; NO_3_^−^, nitrate; SO_4_^2−^, sulfate; SO_2_, sulfur dioxide; NO_2_, nitrogen dioxide.

The characteristics of the study population are summarized in Table 1; Fig. 4. In the cross-sectional study (CHARLS 2015), out of the 10,231 participants included, 48.3% were males. The median age (interquartile range) was 60.00 (15.00) years, and the median BMI (interquartile range) was 23.96 (6.12) kg/m^2^. Significant differences in socio-demographic characteristics were observed, except for marital status, drinking status, public insurance, and PAV, when compared with the relatively healthy participants. Specifically, the multi-morbidity groups were older, had poorer health status, were more prevalent in western regions, and had less sleep time compared to the relatively healthy group. Notably, the respiratory disease group had a lower BMI and a higher proportion of males compared to the healthy group.

In the longitudinal study (CHARLS 2015–2020), the number of MCC cases was 245. Approximately 46.5% of the MCC cases were males. The median age (interquartile range) was 60.00 (13.00) years, and the median BMI (interquartile range) was 23.11 (5.15) kg/m^3^. Most of the MCC participants were married, felt fair, lived in rural villages, had no depression, and had medical insurance. We also identified that 32.2% of the MCC cases lacked education. Significant differences in characteristics such as age, health state, education status, cooking fuel, and depression were observed between non-MCC and MCC cases.

**Table 1:** Characteristics of participants baseline.

Characteristics	Baseline (CHARLS 2015) (*N* = 10,231)				ALL	*P-value*	CHARLS 2015–2020 (*N* = 1938)			
	Respiratory class (*n* = 384)	Stomach-arthritis class (*n* = 934)	Vascular class (*n* = 1701)	Relatively healthy class (*n* = 7192)			MCC (*n* = 245)	Non-MCC (*n* = 1693)	ALL	*P-value*
Age [year, mean (SD)]										
	66.00 (12.50)	62.11 (12.00)	63.00 (13.00)	59.00 (14.00)	60.00 (15.00)	< 0.001***	60.00 (13.00)	57.00 (12.00)	57.00 (13.00)	< 0.001***
Sex [n (%)]										
Male	235 (61.2)	376 (40.3)	782 (46.0)	3548 (49.3)	4941 (48.3)	< 0.001***	114 (46.5)	847 (50.0)	961 (49.6)	0.306
Female	149 (38.8)	560 (59.7)	922 (54.0)	3659 (50.7)	5290 (51.7)		131 (53.5)	846 (45.0)	977 (50.4)	
BMI [kg/m^2^, mean (SD)]										
	23.13 (6.89)	24.16 (6.43)	25.68 (6.02)	23.54 (5.86)	23.96 (6.12)	< 0.001***	23.11 (5.15)	23.09 (4.71)	23.09 (4.72)	0.615
Health state										
Good [n (%)]	48 (12.5)	101 (10.8)	280 (16.5)	2321 (32.3)	2750 (26.9)	< 0.001***	81 (33.1)	830 (49.0)	911 (47.0)	< 0.001***
Fair	177 (45.7)	441 (47.2)	876 (51.5)	3866 (53.8)	5330 (52.1)		123 (50.2)	788 (45.5)	911 (47.0)	
Worse	159 (41.8)	424 (42.0)	548 (32.0)	1020 (14.2)	2151 (21.0)		41 (16.7)	75 (5.5)	116 (6.0)	
Marital status [n (%)]										
Married	380 (99.0)	929 (99.3)	1694 (99.4)	7174 (99.5)	10,177 (99.5)	0.311	243 (99.2)	1684 (99.5)	1927 (99.4)	0.579
Never married	4 (1.0)	7 (0.7)	10 (0.6)	33 (0.5)	54 (0.5)		2 (0.8)	9 (0.5)	11 (0.6)	
Education status [n (%)]										
Illiterate	102 (26.6)	236 (25.2)	361 (21.2)	1633 (22.7)	2332 (22.8)	< 0.001***	79 (32.2)	331 (19.6)	410 (21.2)	< 0.001***
Elementaryor below-school	179 (46.6)	391 (41.8)	635 (37.3)	2812 (39.0)	4017 (39.3)		90 (36.7)	667 (39.4)	757 (39.01)	
Middle school	103 (26.8)	309 (33.0)	708 (41.5)	2762 (38.3)	3882 (37.9)		76 (31.0)	695 (41.1)	771 (39.8)	
Drinking status [n (%)]										
Non-drinker	193 (50.3)	501 (53.5)	950 (55.8)	3881 (53.9)	5525 (54.0)	0.22	134 (54.7)	928 (54.8)	1062 (54.8)	0.972
Drinker	191 (49.7)	435 (46.5)	754 (44.2)	3326 (46.1)	4706 (46.0)		111 (45.3)	765 (45.2)	876 (45.2)	
Smoking status [n (%)]										
Non-smoker	135 (35.2)	512 (54.7)	969 (56.9)	3999 (55.5)	5615 (54.9)	< 0.001***	146 (59.6)	939 (55.5)	1085 (56.0)	0.224
Smoker	249 (64.8)	424 (45.3)	735 (43.1)	3208 (44.5)	4616 (45.1)		99 (40.4)	754 (44.5)	853 (44.0)	
Cooking fuel [n (%)]										
Solid fuel	186 (48.4)	498 (53.2)	613 (36.0)	2965 (41.1)	4262 (41.7)	< 0.001***	125 (51.0)	1024 (60.5)	1149 (59.3)	0.008**
Clean fuel	197 (51.3)	436 (46.6)	1086 (63.7)	4218 (58.5)	593 (58.0)		118 (48.2)	665 (39.3)	783 (40.4)	
No cook	1 (0.3)	2 (0.2)	5 (0.3)	24 (0.3)	32 (0.3)		2 (0.8)	4 (0.2)	6 (0.3)	
Residence [n (%)]										
Urban community	143 (37.2)	370 (39.5)	877 (51.5)	2697 (37.4)	4087 (40.0)	< 0.001***	80 (32.7)	589 (34.8)	669 (34.5)	0.511
Rural village	241 (62.8)	566 (60.5)	827 (48.5)	4510 (62.6)	6144 (60.0)		165 (67.3)	1104 (65.2)	1269 (65.5)	
Regional categories [n (%)]										
East	102 (26.6)	180 (19.2)	580 (34.0)	2645 (36.7)	3507 (34.3)	< 0.001***	99 (40.4)	707 (41.8)	806 (41.6)	0.285
West	80 (20.8)	148 (15.8)	210 (12.3)	1228 (17.0)	1666 (16.3)		52 (21.2)	285 (16.8)	310 (16.0)	
Midland	138 (35.9)	310 (33.1)	563 (33.0)	2380 (33.0)	3391 (33.1)		65 (26.5)	517 (30.5)	582 (30.0)	
Northeast	64 (16.7)	298 (31.8)	351 (20.6)	954 (13.2)	1667 (16.3)		29 (11.8)	184 (10.9)	213 (11.0)	
Public insurance [n (%)]										
No	33 (8.6)	76 (8.1)	172 (10.1)	774 (10.7)	1055 (10.3)	0.31	29 (11.8)	145 (8.6)	174 (9.0)	0.094
Yes	351 (91.4)	860 (91.9)	1532 (89.9)	6433 (89.3)	9176 (89.7)		216 (88.2)	1548 (91.4)	1764 (91.0)	
Depression [n (%)]										
No	301 (78.4)	648 (69.2)	1466 (86.0)	6487 (90.0)	8902 (87.0)	< 0.001***	215 (87.8)	1615 (95.4)	1830 (94.4)	< 0.001***
Yes	83 (21.6)	288 (30.8)	238 (14.0)	720 (10.0)	1329 (13.0)		30 (12.2)	78 (4.6)	108 (5.6)	
Social activity [n (%)]										
No	205 (53.4)	470 (50.2)	775 (45.5)	3641 (50.5)	5091 (49.8)	0.001**	132 (53.9)	855 (50.5)	987 (50.9)	0.323
Yes	179 (46.6)	466 (49.8)	929 (54.5)	3566 (49.5)	5140 (50.2)		113 (46.1)	838 (49.5)	951 (49.1)	
Actual sleep [hours, mean (SD)]										
	6.00 (3.00)	6.00 (3.00)	6.50 (3.00)	7.00 (3.00)	6.00 (3.00)	0.001**	6.00 (3.00)	7.00 (2.00)	7.00 (2.00)	0.077
PAV^c^ [mean (SD)]										
	1592.36 (2006.61)	1659.22 (1881.41)	1731.50 (1837.47)	1662.00 (1930.55)	1672.89 (1916.45)	0.585	1596.41 (2657.06)	1732.50 (2404.244)	1732.50 (2434.50)	0.487

^a^ Mean with standard deviation for continuous data and counts with percentages for categorical variables.

^c^ 3153 data are missing.

Compared to no relatively healthy class, significant at *P < 0.05, **P < 0.01 and ***P < 0.001

**Table 2:** Descriptive statistics for the average measurements.

	Mean	Std.	min	max	P25	P50	P75	IQR
Air pollution								
PM_2.5_ (µg/m^3^)	58.48	17.94	27.85	97.37	43.71	56.23	71.21	27.49
PM_1_ (µg/m^3^)	32.48	9.82	12.75	53.26	25.71	31.47	39.29	13.58
PM_10_ (µg/m^3^)	99.10	30.50	53.78	163.94	76.81	96.70	121.95	45.13
O_3_ (µg/m^3^)	84.35	8.26	63.40	102.22	77.70	83.42	90.87	13.18
NO_2_ (µg/m^3^)	30.87	9.31	14.64	49.33	24.07	30.18	37.18	13.11
SO_2_ (µg/m^3^)	32.53	14.02	12.46	65.46	21.88	28.04	40.78	18.90
CO (mg/m^3^)	1.22	0.33	0.55	2.04	0.95	1.14	1.40	0.46
Cl^−^ (µg/m^3^)	2.41	0.92	0.96	4.36	1.71	2.26	3.02	1.30
NH_4_^+^ (µg/m^3^)	7.57	2.23	3.79	11.40	5.62	7.38	9.45	3.83
NO_3_^−^ (µg/m^3^)	10.56	3.78	4.68	17.20	6.97	10.15	14.80	7.83
SO_4_^2−^ (µg/m^3^)	11.98	3.17	5.44	18.10	9.47	12.11	14.28	4.81
Temperature (°C)	13.75	5.07	1.28	22.79	10.91	14.93	16.34	
Humidity (%)	69.06	8.16	44.62	81.17	63.17	70.09	75.94	

Notes: Abbreviations: P25, P50, P75, Lower, middle and higher quartiles of variables; IQR, inter-quartile range; PM_2.5_, particle with aerodynamic diameter ≤ 2.5 μm; PM_1_, particle with aerodynamic diameter ≤ 1 μm; PM_10_, particle with aerodynamic diameter ≤ 10 μm; CO, carbonic oxide; O_3_, ozone; Cl^−^, chloride; NH_4_^+^, ammonium; NO_3_^−^, nitrate; SO_4_^2−^, sulfate; SO_2_, sulfur dioxide; NO_2_, nitrogen dioxide.

**Fig. 5:**
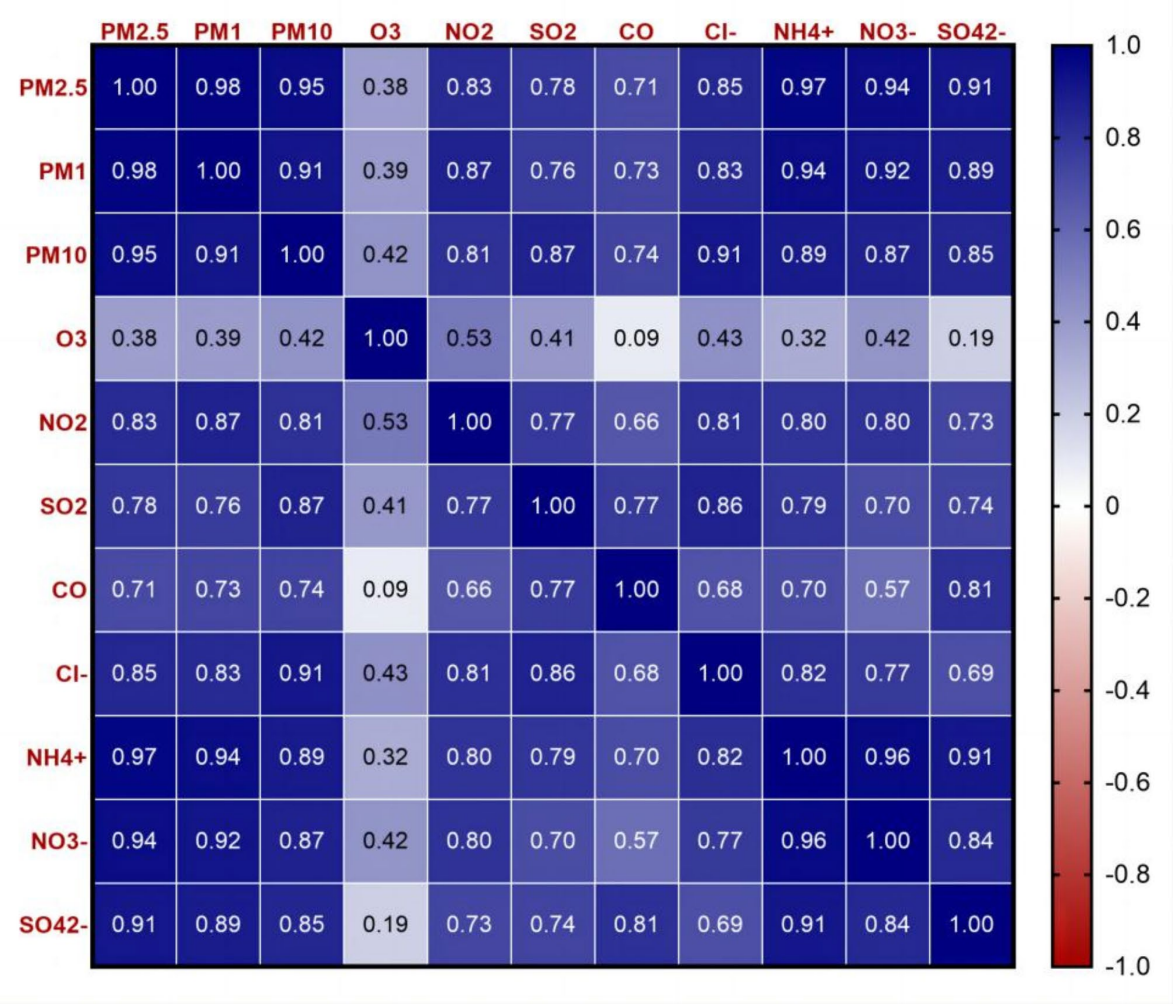
The Pearson correlation coefficients between the air pollutants.

According Table 2, the levels of exposure in the environment over the past five years were PM_2.5_ (58.48 µg/m^3^), PM_1_, PM_10_, O_3_, NO_2_, SO_2_, CO, Cl^−^, NH_4_^+^, NO_3_^−^, and SO_4_^2−^ 58.48 µg/m^3^, 32.48 µg/m^3^, 99.10 µg/m^3^, 84.35 µg/m^3^, 30.87 µg/m^3^, 35.53 µg/m^3^, 1.22 mg/m^3^, 2.41 µg/m^3^, 7.57 µg/m^3^, 10.56 µg/m^3^, and 11.98 µg/m^3^, respectively. The corresponding correlation coefficients (r) vary from 0.70 to 0.98 (Table S4 and Fig. 5).

### Association between air pollution and MCC patterns

**Fig. 6:**
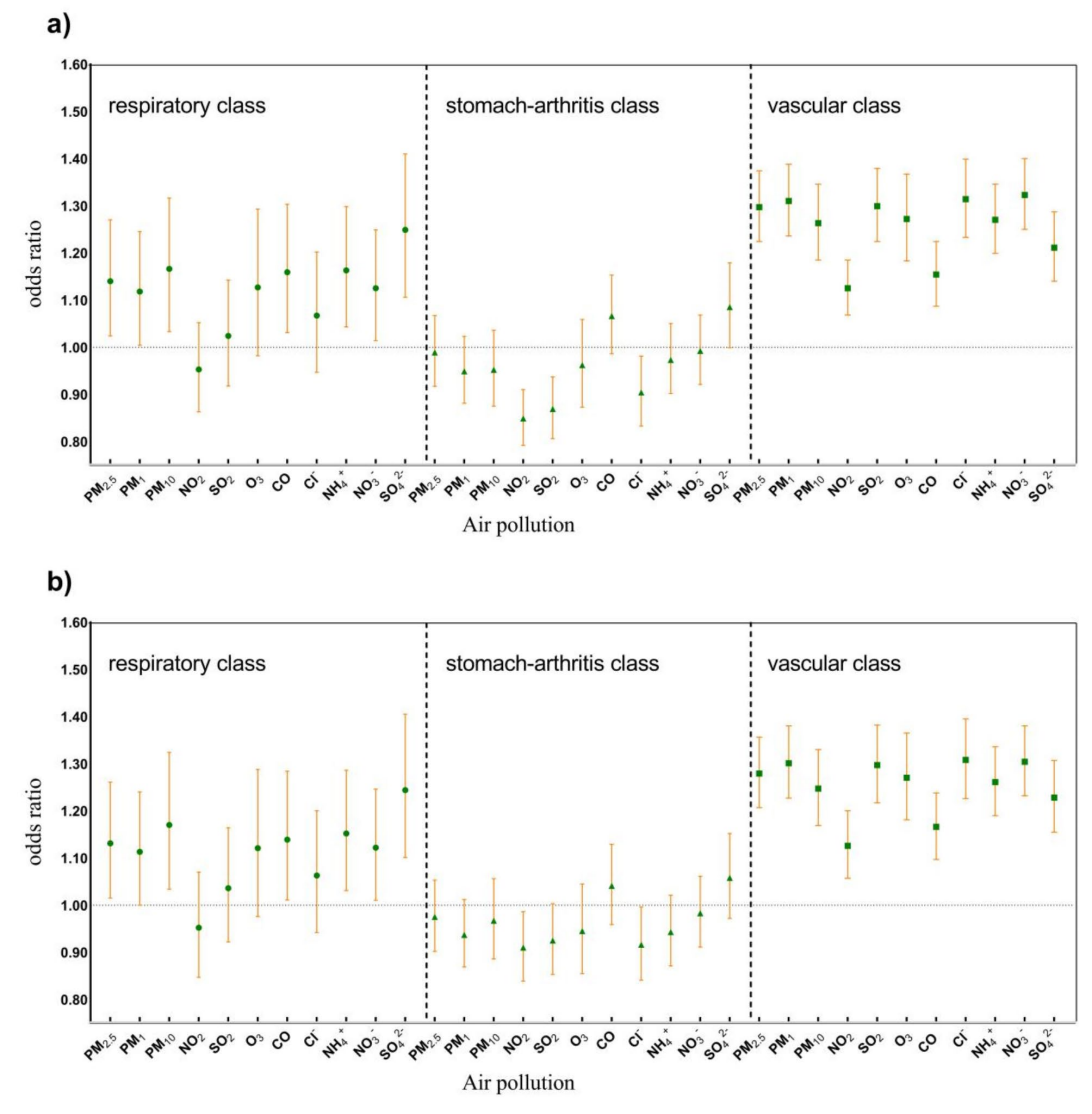
OR (95% CI) of the correlation between 3-year average air pollutants and MCC patterns: a Model 1 adjusted age, married status, education state, sex and health status. b Model 2 also adjusted for patients’ BMI, lifestyle habits (including alcohol consumption, smoking), cooking fuel, place of residence, economic region category, public insurance, depression, physical activity, social activity, and meteorological factors (temperature, relative humidity).

The incidence of chronic respiratory diseases was significantly increased with each additional quartile of PM_2.5_, PM_10_, CO, NH_4_^+^, NO_3_^−^, and SO_4_^2−^, with the corresponding odds ratios (OR) and 95% CI of 1.131 (95% CI: 1.015,1.261), 1.170 (95% CI: 1.034,1.324), 1.139 (95% CI: 1.011,1.284), 1.139 (95% CI: 1.011,1.284), 1.152 (95% CI: 1.031,1.286), 1.122 (95% CI: 1.010,1.246), 1.244 (95% CI: 1.101,1.405). Further, with each increase of one quartile in PM_2.5_, PM_1_, PM_10_, NO_2_, SO_2_, O_3_, CO, Cl^−^, NH_4_^+^, NO_3_^−^, and SO_4_^2−^ in the environment, a notable rise in the occurrence of cardiovascular illnesses, with the corresponding OR (95% CI) of 1.279 (95% CI: 1.207, 1.356), 1.301 (95% CI: 1.227, 1.380), 1.247 (95% CI: 1.169, 1.330), 1.126 (95% CI: 1.057, 1.200), 1.297 (95% CI: 1.217, 1.382), 1.270 (95% CI: 1.181, 1.365), 1.166 (95% CI: 1.097, 1.238), 1.308 (95% CI: 1.226, 1.395), 1.261 (95% CI: 1.190, 1.336), 1.304 (95% CI: 1.232, 1.380), and 1.228 (95% CI: 1.155, 1.307). It is noteworthy that the rise in O_3_ and Cl^−^ concentrations is linked to a decrease in the incidence of gastric joint-related diseases (Fig. 6 and Table S5).

### Association between air pollution and the risk of MCC

**Fig. 7:**
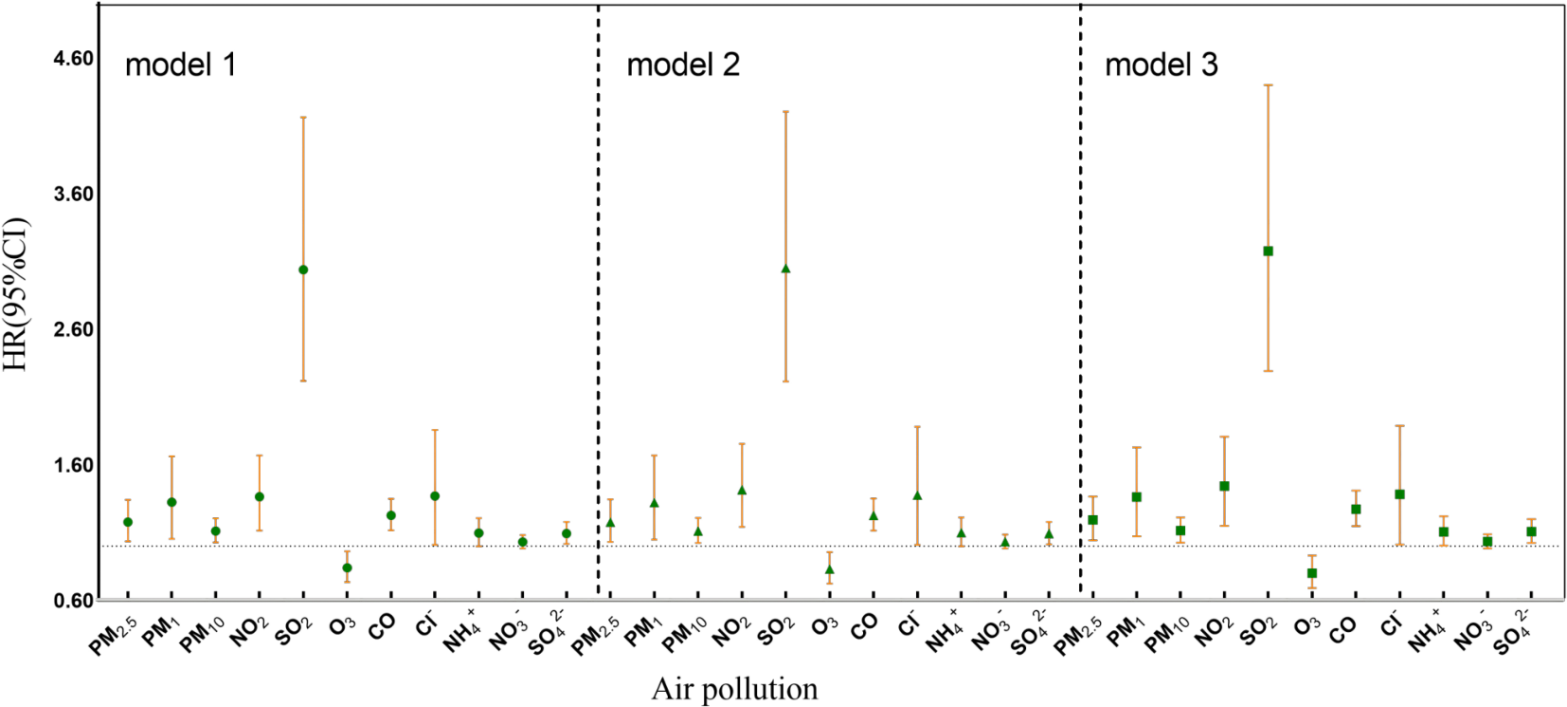
HRs (95% CI) for the association between 3-year average air pollution and the risk of MCC. *Notes*: Abbreviations: HR, hazard risk; 95%CI, 95% confidence intervals.

In the present study, it was observed that, after adjusting for multiple factors, with a 10 µg/m^3^ rise in PM_2.5_, PM_1_, PM_10_, O_3_, NO_2_, and SO_2,_ the HRs (95% CI) for developing MCC were: 1.194 (95% CI: 1.043, 1.367), 1.362 (95% CI: 1.073, 1.728), 1.115 (95% CI: 1.026, 1.212), 0.901 (95% CI: 0.690, 0.930), 1.443 (95% CI: 1.151, 1.808), and 3.175 (95% CI: 2.291, 4.401). Specifically, for every 0.1 mg/m^3^ rise in CO, HRs (95% CI) for developing MCC were 1.272 (95% CI: 1.149, 1.410). Similarly, for Cl^−^, NH_4_^+^, NO_3_^−^, and SO_4_^2−^, each 1 µg/m^3^ rise was correlated to the HRs (95% CI) values of 1.382 (95% CI: 1.011, 1.888), 1.107 (95% CI: 1.003, 1.222), 1.035 (95% CI: 0.984, 1.088), and 1.108 (95% CI: 1.023, 1.200) (Table 3; Fig. 7). These findings underscored the significance of limiting and reducing environmental pollution if the risk of chronic diseases has to be mitigated.

**Table 3:** HRs (95% CI) for the association between 3-year average air pollution and the risk of MCC.

Pollutants	Model 1	Model 2	Model 3
PM_2.5_	1.178 (1.034, 1.342)	1.178 (1.032, 1.346)	1.194 (1.043, 1.367)
PM_1_	1.324 (1.054, 1.663)	1.322 (1.048, 1.668)	1.362 (1.073, 1.728)
PM_10_	1.112 (1.027, 1.205)	1.112 (1.024, 1.208)	1.115 (1.026, 1.212)
NO_2_	1.364 (1.115, 1.668)	1.415 (1.142, 1.754)	1.443 (1.151, 1.808)
SO_2_	3.038 (2.217, 4.163)	3.051 (2.214, 4.204)	3.175 (2.291, 4.401)
O_3_	0.840 (0.734, 0.961)	0.832 (0.724, 0.956)	0.801 (0.690, 0.930)
CO	1.227 (1.117, 1.349)	1.228 (1.116, 1.351)	1.272 (1.149, 1.410)
Cl^−^	1.370 (1.010, 1.857)	1.378 (1.009, 1.880)	1.382 (1.011, 1.888)
NH_4_^+^	1.098 (0.998, 1.207)	1.100 (0.998, 1.212)	1.107 (1.003, 1.222)
NO_3_^−^	1.031 (0.983, 1.082)	1.033 (0.983, 1.086)	1.035 (0.984, 1.088)
SO_4_^2−^	1.094 (1.016, 1.180)	1.093 (1.013, 1.179)	1.108 (1.023, 1.200)

*Notes*: Impact estimates have been calculated for each quartile increment in the 3-year mean concentration of air contaminants.

Adjusted:(1)model 1: age, sex, meteorological factors (temperature, relative humidity); (2)model 2: Base mode l + marital status, education state, health status, patients’ BMI, place of residence, social activity, public insurance; (3)model 3: mode 2 + lifestyle habits (including alcohol consumption, smoking), cooking fuel, economic region category, depression, physical activity.

Air pollutants PM_2.5_, PM_1_, PM_10_, NO_2_, SO_2_ (increasing by 10 µg/m^3^ each), CO (increasing by 0.1 mg/m^3^), and PMC (Cl^-^, NH_4_^+^, NO_3_^-^, SO_4_^2-^) (increasing by 1 µg/m^3^ each) are used to calculate the impact estimate.

**Fig. 8:**
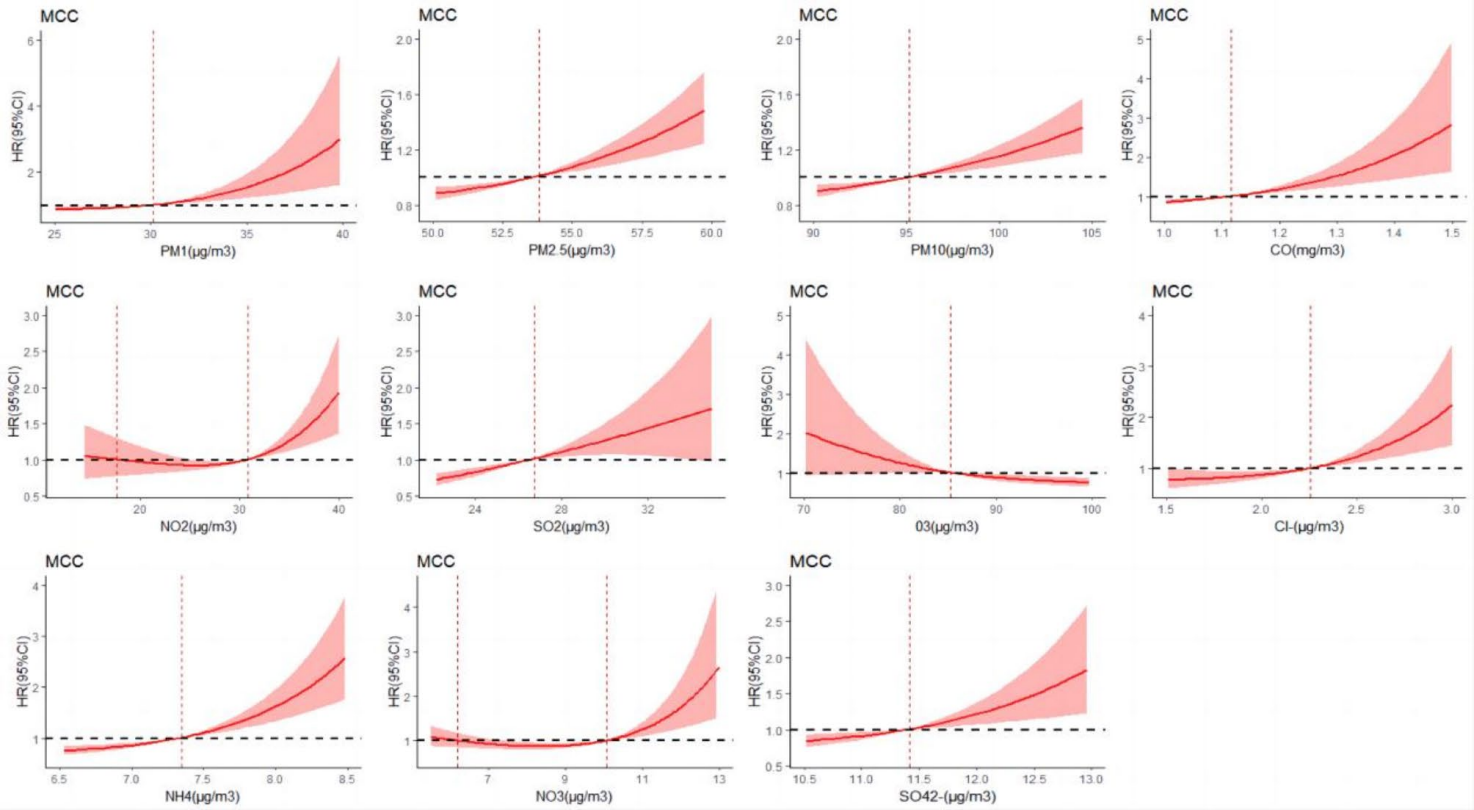
Dose-response relationships of various air pollutants to MCC.

Furthermore, the results revealed a J-shaped relationship of PM_2.5_, PM_1_, PM_10_, SO_2_, CO, Cl^−^, NH_4_^+^, NO_3_^−^, and SO_4_^2−^ with the presence of MCC. Accordingly, the individuals exposed to higher levels of the above pollutants were at an increased risk of developing MCC (Fig. 8).

### Association between air pollution mixture and MCC based on quantile g-computation

**Fig. 9:**
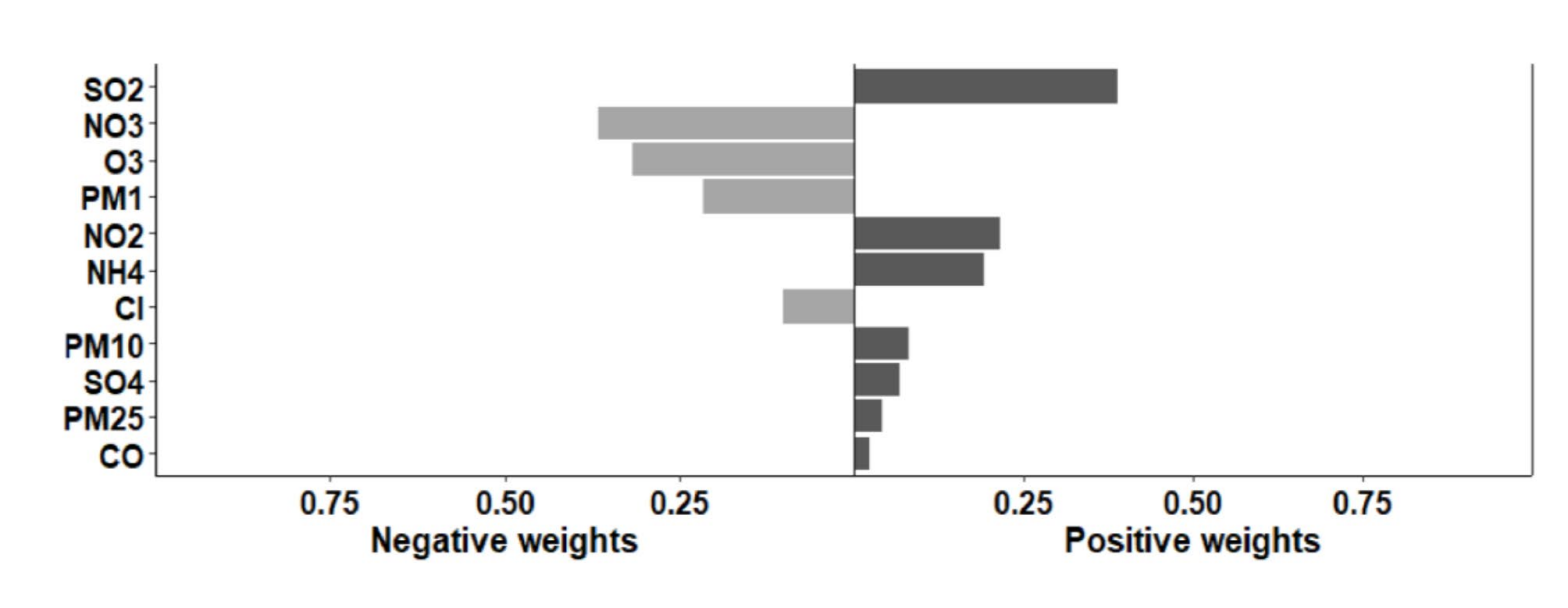
Results from quantile g-computational 11 air pollution mixture analysis.

The component with the highest weight was SO_2_ (weight 0.388), indicating that the effects of combined exposure to air pollutants were the most influential. Co-exposure to the four mixtures modeled, where 11 air pollution mixtures were positively associated with MCC occurrence and cardiovascular disease patterns, with β (95% CI) of 1.083 (95% CI: 0.659, 1.508), and 0.159 (95% CI: 0.075, 0.252), respectively. The results were similar to the co-exposure effects of the other three mixtures (Table 4; Fig. 9).

**Table 4:** Mixture effects on MCC for one quartile increase in the air pollution mixture based on quantile-based g computation.

Model	Respiratory class [β (95% CI)] ^ab^	Stomach-arthritis class [β (95% CI)]^ab^	Vascular class [β (95% CI)]^ab^	MCC [β (95% CI)]^b^
11 mixtures	−0.006 (−0.160, 0.148)	−0.041 (−0.148, 0.066)	0.159 (0.075, 0.252)	1.083 (0.659, 1.508)
Particulate matter	0.143 (0.043, 0.243)	−0.0168 (−0.089, 0.056)	0.196 (0.142, 0.250)	0.351 (0.095, 0.606)
7 major mixtures	0.007 (−0.139, 0.153)	−0.078 (−0.178, 0.023)	0.160 (0.079, 0.240)	1.164 (0.762, 1.566)
PMCs	0.099 (−0.010, 0.209)	0.009 (−0.067, 0.084)	0.239 (0.183, 0.295)	0.542 (0.262, 0.821)

^a^ β (95% CI)contrast to the relative-healthy class

^b^ adjusted for patients’ BMI, lifestyle habits (including alcohol consumption, smoking), cooking fuel, place of residence, economic region category, public insurance, depression, physical activity, social activity, and meteorological factors (temperature, relative humidity

Abbreviations: qg-computation, quantile-based g-computation; CI, confidence interval;

(1) 11 mixtures: PM_1_, PM_10_, PM_2.5_, CO, O_3_, NO_2_, SO_2_, NO_3_^−^, Cl^−^, NH_4_^+^, and SO_4_^2−^

(2) particulate matter: the mixtures for PM_1_, PM_10_, PM_2.5_

(3) 7 major mixtures: the mixtures for PM_1_, PM_10_, PM_2.5_, CO, O_3_, NO_2_, SO_2_

(4) PMCs: PM_2.5_ chemical composition the mixtures for NO_3_^−^, Cl^−^, NH_4_^+^, and SO_4_^2−^

### Interaction effects analyses

Significant interactions of PM_1_, PM_10_, PM_2.5_, CO, O_3_, NO_2_, SO_2_, NO_3_^−^, Cl^−^, NH_4_^+^, and SO_4_^2−^ with gender, age, drinking, smoking, and health status on the prevalence of MCC and its patterns were identified. For example, results showed a significantly increased prevalence of stomach-related/arthritic diseases for males (OR = 1.101, 95% CI: 1.049, 1.252) but not for females (OR = 1.018, 95% CI: 0.962, 1.077) (P-interaction = 0.048), each additional quartile increase in SO_4_^2−^. For females, an increase in Cl^−^ is associated with the prevalence of stomach-related/arthritic diseases. Concurrently, for air pollutants such as PM_1_, PM_2.5_, O_3_, NO_2_, SO_2_, NO_3_^−^, NH_4_^+^, and SO_4_^2−^, stomach-related/arthritic diseases were found to be stronger in younger individuals aged more than 60. This indicates that, within this age group, higher concentrations of these pollutants were associated with a higher prevalence of these diseases. Specifically, we identified that NO_2_ had stronger effects on stomach-related/arthritic diseases in people with poor/very poor health status than those with good/very good health status, indicating a modification effect of health status. We also identified that PM_2.5_ had stronger effects on vascular diseases in people who had a habit of drinking. The results from the simple stratified analyses were similar to those from the interaction effect analyses. (Table S6-9).

## Discussion

### Main findings

This study represents the first statewide epidemiological investigation in China to explore the relationship between continuous exposure to air pollution and Multiple Chronic Conditions (MCC), with a particular focus on the moderating effects of gender, age, health status, smoking, and alcohol consumption. Our cross-sectional analysis revealed significant associations between specific air pollutants and MCC patterns. Furthermore, our longitudinal analysis revealed significant associations between specific air pollutants and the prevalence of MCC. Notably, our findings revealed that gender, age, general health status, and alcohol consumption emerged as key modulators. These results contribute to a deeper understanding of the complex interplay between air pollution and MCC, underscoring the need for targeted interventions and policies to alleviate the burden of air pollution on vulnerable populations.

### Comparison with other studies

Our study uniquely employed Latent Class Analysis (LCA) to classify clinically significant MCC subgroups, an approach that provides valuable insights for identifying high-risk elderly populations with multiple diseases. The method has proven effective in promoting early interventions that can mitigate chronic disease progression and reduce the associated mortality^43^. Similar to previous studies, our analysis identified specific clustering patterns among chronic diseases, particularly respiratory and cardiovascular diseases, which were significantly associated with air pollution exposure. These results echo findings from earlier studies, confirming that air pollution, especially particulate matter (PM_2.5_, CO, PM_10_), is a major contributor to respiratory and cardiovascular conditions.

Interestingly, while our study revealed significant correlations between several air pollutants (e.g., PM_2.5_, SO_2_) and the incidence of respiratory diseases, we did not observe significant associations with NO_2_, SO_2_, and O_3_ as previously reported in other studies. For instance, prior research has highlighted the relevance of NO_2_ to respiratory disease hospitalization^44^ and the impact of SO_2_ and O_3_ on increased mortality from chronic obstructive pulmonary disease (COPD) and pneumonia. These discrepancies may stem from differences in study design, population characteristics, and geographical contexts. Given the potential public health implications, further experimental or meta-analytical studies are warranted to confirm these associations, particularly in the Chinese context.

Moreover, our findings on the relationship between air pollution and stomach-arthritis diseases revealed no significant or inverse associations, contrary to some earlier research. For example, Adami et al. reported that exposure to pollutants like PM_2.5_, NO_2_, PM_10_, O_3_, and CO increased the risk of arthritic flare-ups^45^. Similarly, Alsaber et al. found that arthritis risk was associated with higher levels of SO_2_ and NO_2_, though not with PM_10_, O_3_, or CO^46^. Additionally, Chang et al. discovered an increased likelihood of developing rheumatoid arthritis (RA) among individuals exposed to PM_2.5_ and NO_2_^47^. In contrast, research on digestive system diseases remains limited. However, a study from the Ningbo cohort in China identified a significant correlation between exposure to air contaminants (PM_2.5_, O_3_, CO, SO_2_, and NO2) and hospitalization for digestive diseases^48^. The inconsistencies among these studies may be attributed to several factors, including differences in geographic locations, study populations, exposure measurement methods, exposure durations, and outcome definitions used across studies. This variability underscores the need for further research to determine whether air pollution is a contributing factor to stomach-related or arthritis disorders. Notably, our study is one of the most comprehensive in evaluating the effects of 11 different air pollutants on MCC. Our longitudinal study, adjusted for relevant factors, revealed that the prevalence of MCC increased with exposure to pollutants such as PM_2.5_, PM_1_, PM_10_, NO2, SO_2_, CO, Cl^-^, NH_4_^+^, and SO_4_^2-^. A significant finding was that an increase of 10 µg/m^3^ in SO_2_ was associated with a HRs (95% CI) of 3.175 (95% CI: 2.291, 4.401) for MCC. However, no correlation was noted with NO_3_^-^, and a negative correlation was observed with O_3_. Due to the limited number of relevant epidemiological studies, few comparisons are available, highlighting the need for further in-depth research to confirm these findings.

###  Potential biological mechanisms

The mechanisms linking air pollution to Multiple Chronic Conditions (MCC) are complex but involve several key pathways. Prolonged exposure to air pollutants induces oxidative stress, inflammation, and immune activation, which are central to the development of chronic diseases^49^. Pollutants like PM_2.5_ can enter the bloodstream, triggering inflammation that contributes to atherosclerosis and other conditions^50^. Air pollutants also disrupt normal cellular functions, including pulmonary surfactant activity^51^ and alveolar macrophage clearance^52^, leading to respiratory and cardiovascular issues. The oxidative stress caused by pollutants generates reactive oxygen species (ROS), which damage cells and contribute to chronic diseases^53,54^. Additionally, air pollution can interfere with gene expression^55^ and disrupt metabolic pathways^56,57^, leading to insulin resistance, liver stress, and the progression of type 2 diabetes. These disruptions can result in abnormal cell function, potentially contributing to the progression of chronic diseases. Pollutants also activate metalloproteinases, increasing the risk of heart attacks and strokes^58^. Additionally, the immune system may overreact to pollutants, causing chronic inflammatory conditions like atherosclerosis and COPD^59^.

### Policy implications

Our study has several important implications for various stakeholders. To mitigate the health burden of air pollution, especially among vulnerable groups such as the elderly and individuals with pre-existing health conditions, a multifaceted approach is necessary.

First, air quality alert systems tailored to these populations^60^, coupled with public health education campaigns^61^, can empower individuals to take proactive measures to reduce their exposure. Policymakers, healthcare providers, and community organizations must collaborate to ensure timely dissemination of this information. Second, integrating air quality data into clinical decision-making processes can enhance personalized medicine^62^. By considering local air pollution levels in treatment plans, healthcare professionals can better assess patient risks and implement targeted interventions. Third, stricter air quality standards in areas frequented by vulnerable populations, along with targeted mitigation measures such as air purification systems, should be prioritized^63^. Lastly, ongoing research is crucial to deepen our understanding of the health impacts of air pollution, thereby informing more effective prevention and intervention strategies^64^. A holistic approach encompassing education, policy reform, clinical integration, and research is essential to protect vulnerable populations from the detrimental effects of air pollution.

### Strengths and limitations

This study is one of the most comprehensive in China, utilizing both cross-sectional and longitudinal analyses to examine the individual and combined effects of air pollutants on MCC prevalence and patterns. The use of nationally representative data from the China Health and Retirement Longitudinal Study (CHARLS) enhances the external validity of our findings. Additionally, employing satellite remote sensing technologies and advanced statistical models improved the accuracy of air pollutant measurements, offering new scientific insights into the health impacts of air pollution.

However, this study has limitations. The high correlation between various pollutants complicates the assessment of their individual effects. The reliance on self-reported data may introduce recall bias, particularly among older participants. Furthermore, unmeasured confounding factors, such as diet, salt intake, and other environmental variables (e.g., green spaces, noise), were not considered. The study also measured community-level rather than individual exposure to air pollution, which may limit the precision of our findings. Lastly, since the study focused exclusively on middle-aged and older adults in China, caution is needed when generalizing the results to other populations. Further research is necessary to validate our conclusions across different demographics and settings.

## Conclusions

This study is one of the few that examines the relationship between air pollution and Multiple Chronic Conditions (MCC) among middle-aged and elderly Chinese individuals. The findings indicate that prolonged exposure to high levels of air pollutants is strongly associated with the prevalence and patterns of MCC. Factors such as gender, age, alcohol consumption, and overall health status can influence these effects. To protect those most vulnerable to MCC, it is crucial to implement advanced preventive strategies. The study recommends a multifaceted approach, including air quality alert systems tailored to these populations, integration of air quality data into clinical decisions, prioritized policy interventions, and continued research to refine and apply effective prevention methods.

## Electronic supplementary material

Below is the link to the electronic supplementary material.

Supplementary Material 1

## Data Availability

Data will be made available on request. Please contact the corresponding author if you would like a copy of the data from this study.
