# Supplementary material for: Individual and joint exposure to air pollutants and patterns of multiple chronic conditions
Source: Sci Rep. 2024 Sep 30;14:22733. doi: 10.1038/s41598-024-73485-7 (PMC11443143; doi:10.1038/s41598-024-73485-7)
Supplement: Supplementary file 1 — Supplementary Material 1 [file 41598_2024_73485_MOESM1_ESM.pdf]

Supplementary Material

Individual and Joint Exposure to Air Pollutants and Patterns of Multiple Chronic Conditions

Contents

Table/Figure		Page
**Figure S1.**	Sensitivity analyses of long-term exposure to air pollutants and MCC patterns.	2-3
**Figure S2.**	Sensitivity analyses of long-term exposure to air pollutants and risk of MCC.	3
**Table S1.**	Coefficient of determination for cross-validation (CV-R2) and root mean square error (RMSE) for air pollution.	4
**Table S2.**	Comparison of the fit statistics of models of LCA	4
**Table S3.**	Item response probability from four-class model	5
**Table S4.**	The Pearson correlation coefficients among air pollutants	5
**Table S5.**	HRs and 95 % CI for the association between air pollution and MCC patterns	6-7
**Table S6.**	Results of the stratified analyses.(Cross-sectional study)	8-12
**Table S7.**	Results of the stratified analyses.(Longitudinal study)	13-15
**Table S8.**	Results of the interaction analyses.(Cross-sectional study)	16-20
**Table S9.**	Results of the interaction analyses.(Longitudinal study)	21-23
**Table S10.**	Sensitivity analyses of air pollutants and MCC patterns(Cross-sectional study)	24-25
**Table S11.**	Sensitivity analyses of air pollutants and risk of MCC (Longitudinal study)	26

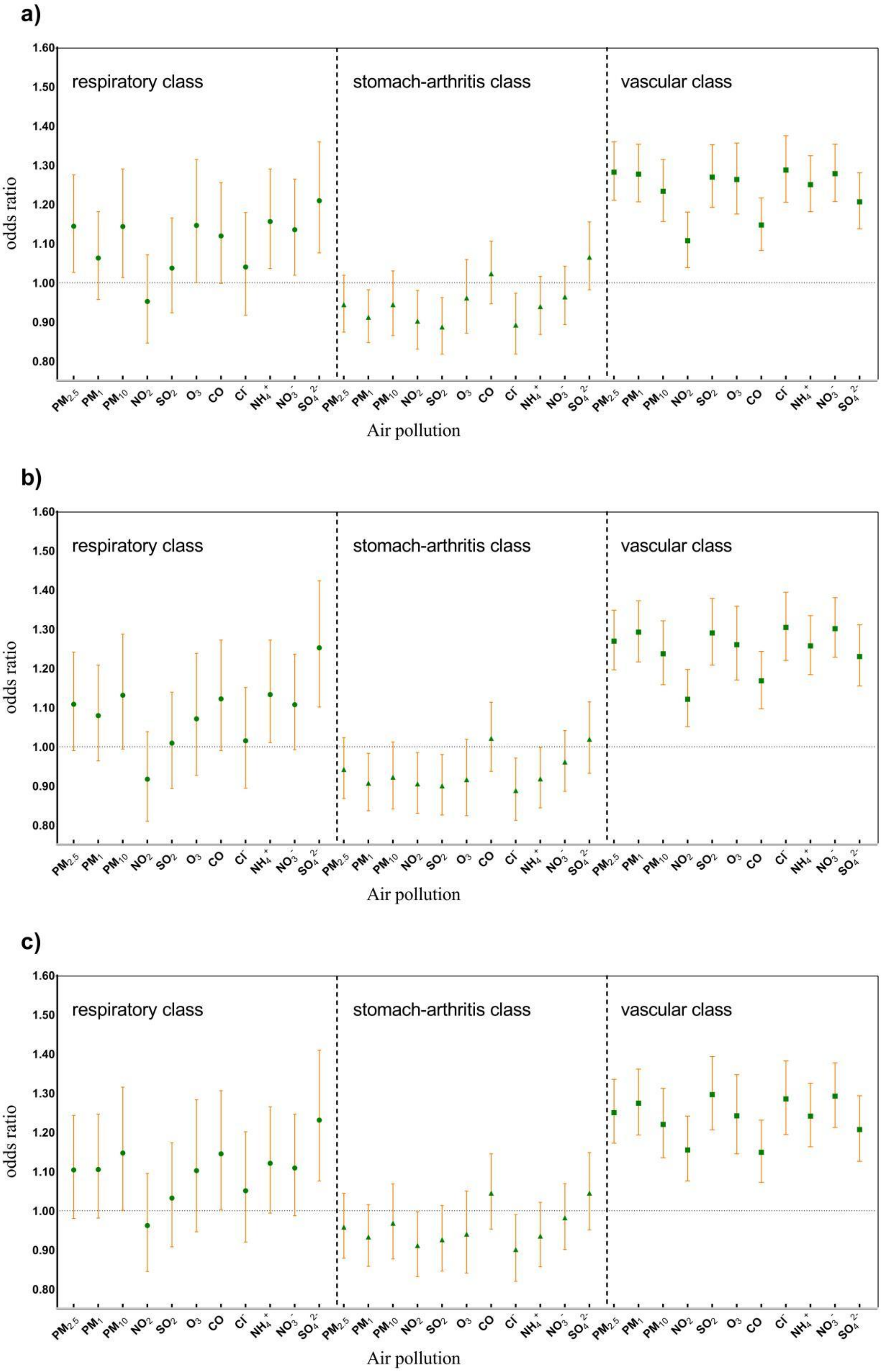

Figure S1. Sensitivity analyses of long-term exposure to air pollutants and MCC patterns.

Notes: Abbreviations:odds ratio: OR; 95% confidence Interval: 95% CI;

In Group 2 and Group 3 ,impact estimates have been calculated for each quartile increment in the 3-year mean concentration of air contaminants.

a)Group 1: The exposure concentrations of air pollutants are two-year averaged.

b)Group 2: Excluded participants in very poor health.

c)Group 3: Excluded missing values for age,bmi, place of residence,public insurance,social activity, and physical activity level at baseline

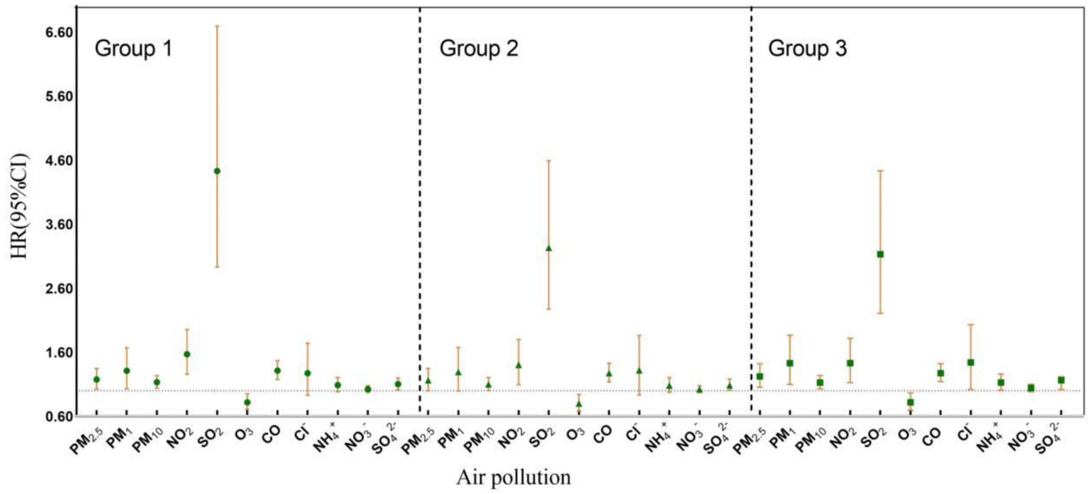

Figure S2. Sensitivity analyses of long-term exposure to air pollutants and risk of MCC.

Notes:Abbreviations:RR, relative risk; 95%CI, 95% confidence intervals.

In Group 2 and Group 3 ,impact estimates have been calculated for each quartile increment in the 3-year mean concentration of air contaminants.

a)Group 1: The exposure concentrations of air pollutants are two-year averaged.

b)Group 2: Excluded participants in very poor health.

c)Group 3: Excluded missing values for age,bmi, place of residence,public insurance,social activity, and physical activity level at baseline

Table S1. Coefficient of determination for cross-validation (CV-R2) and root mean square error (RMSE) for air pollution.

Pollutants	CV-R2	RMSE
$PM_{2.5}$	0.92	10.76 μg/m^3
PM_1	0.83	9.50 μg/m^3
PM_{10}	0.90	21.12 μg/m^3
NO_2	0.84	7.99 μg/m^3
SO_2	0.84	10.07 μg/m^3
O_3	0.89	15.77 μg/m^3
CO	0.80	0.29 mg/m^3
Cl^-	0.66	2.3 μg/m^3
NH_4^+	0.71	4.3 μg/m^3
NO_3^-	0.75	6.6 μg/m^3
SO_4^{2-}	0.74	6.0 μg/m^3

Notes:Abbreviations: $PM_{2.5}$, particle with aerodynamic diameter ≤2.5 μm; PM_1, particle with aerodynamic diameter ≤1 μm; PM_{10}, particle with aerodynamic diameter ≤10 μm; SO_2, sulfur dioxide; NO_2, nitrogen dioxide; CO, carbonic oxide; O_3, ozone; Cl^-, chloride; NH_4^+, ammonium; NO_3^-, nitrate; SO_4^{2-}, sulfate;

Table S2. A Comparison of the fit statistics of models of LCA

n(2-6)	AIC	BIC	aBIC	Entropy	VLMR-LRT	BLRT	class		
2	94528	94738	94646	0.585	0	0	1	2916	0.285
							2	7315	0.715
3	93521	93840	93700	0.669	0.0012	0	1	1031	0.101
							2	2068	0.202
							3	7132	0.697
**4**	**93066**	**93494**	**93306**	**0.718**	**0.0001**	**0**	**1**	**384**	**0.038**
							**2**	**936**	**0.091**
							**3**	**1704**	**0.167**
							**4**	**7207**	**0.704**
5	92852	93389	93154	0.634	0.0001	0	1	1776	0.174
							2	336	0.033
							3	1575	0.154
							4	498	0.049
							5	6046	0.591
6	92794	93439	93156	0.646	0.2485	0	1	480	0.047
							2	185	0.018
							3	1249	0.122
							4	344	0.034
							5	6467	0.632
							6	1506	0.147

Table S3. Item response probability from four-class model

Chronic conditions	Total (n=10231)	respiratory class (n = 384)	stomach-arthritis class (n = 934)	vascular class (n =1701)	relatively healthy class (n = 7192)
		Latent class			
Hypertension	0.3335	0.4089	0.5032	0.8527	0.1847
Diabetes	0.1013	0.1016	0.1496	0.3932	0.0259
Cancer	0.0162	0.0208	0.0502	0.0158	0.0117
Chronic lung disease	0.1249	0.9974	0.3547	0.0540	0.0654
Chronic heart diseases	0.1826	0.4063	0.6784	0.4613	0.0404
Stroke	0.0334	0.0365	0.0630	0.1291	0.0068
Emotional problems	0.0165	0.0286	0.0694	0.0158	0.0092
Arthritis	0.3890	0.5000	0.8579	0.3492	0.3316
Dyslipidemia	0.1943	0.1406	0.3729	0.6714	0.0612
Chronic liver disease	0.0605	0.0677	0.2970	0.0540	0.0309
Kidney disease	0.0867	0.0755	0.4220	0.0745	0.0466
Stomach disease	0.2874	0.2865	0.8387	0.2025	0.2359
Asthma	0.0550	0.8854	0.1282	0.0076	0.0125
Memory-related diseases	0.0240	0.0208	0.0823	0.0581	0.0086

Table S4. The Pearson correlation coefficients among air pollutants

	$PM_{2.5}$	PM_1	PM_{10}	O_3	NO_2	SO_2	CO	CL^-	$NH4^+$	$NO3^-$	$SO4^{2-}$
$PM_{2.5}$	1.00										
PM_1	0.98	1.00									
PM_{10}	0.95	0.91	1.00								
O_3	0.38	0.39	0.42	1.00							
NO_2	0.83	0.87	0.81	0.53	1.00						
SO_2	0.78	0.76	0.87	0.41	0.77	1.00					
CO	0.71	0.73	0.74	0.09	0.66	0.77	1.00				
CL^-	0.85	0.83	0.91	0.43	0.81	0.86	0.68	1.00			
$NH4^+$	0.97	0.94	0.89	0.32	0.80	0.79	0.70	0.82	1.00		
$NO3^-$	0.94	0.92	0.87	0.42	0.80	0.70	0.57	0.77	0.96	1.00	
$SO4^{2-}$	0.91	0.89	0.85	0.19	0.73	0.74	0.81	0.69	0.91	0.84	1.00

Notes:Abbreviations: $PM_{2.5}$, particle with aerodynamic diameter ≤2.5 μm; PM_1, particle with aerodynamic diameter ≤1 μm; PM_{10}, particle with aerodynamic diameter ≤10 μm; SO_2, sulfur dioxide; NO_2, nitrogen dioxide; CO, carbonic oxide; O_3, ozone; Cl^-, chloride; $NH4^+$, ammonium; $NO3^-$, nitrate; $SO4^{2-}$, sulfate;

Table S5. HRs and 95 % CI for the association between air pollution and MCC patterns

Class group	pollution (*IQR*)	Model 1 OR and 95%CI	*P-value*	Model 2 OR and 95%CI	*P-value*
respiratory class (n=384)	$PM_{2.5}$	1.140(1.024,1.270)	0.017*	1.131(1.015,1.261)	0.025*
	PM_1	1.118(1.004,1.245)	0.041*	1.113(1.000,1.240)	0.051
	PM_{10}	1.166(1.033,1.316)	0.013*	1.170(1.034,1.324)	0.013*
	O_3	0.953(0.863,1.052)	0.337	0.952(0.847,1.070)	0.41
	NO_2	1.024(0.918,1.142)	0.667	1.036(0.922,1.164)	0.552
	SO_2	1.127(0.982,1.293)	0.089	1.121(0.976,1.288)	0.105
	CO	1.159(1.031,1.303)	0.013*	1.139(1.011,1.284)	0.033*
	Cl^-	1.067(0.947,1.202)	0.285	1.063(0.942,1.200)	0.32
	$NH4^+$	1.163(1.043,1.298)	0.007**	1.152(1.031,1.286)	0.012*
	$NO3^-$	1.125(1.014,1.249)	0.026*	1.122(1.010,1.246)	0.032*
	$SO4^{2-}$	1.249(1.106,1.410)	<0.001***	1.244(1.101,1.405)	<0.001***
stomach-arthritis class (n=934)	$PM_{2.5}$	0.989(0.917,1.067)	0.779	0.975(0.902,1.053)	0.515
	PM_1	0.949(0.881,1.023)	0.171	0.937(0.869,1.012)	0.096
	PM_{10}	0.952(0.875,1.036)	0.256	0.967(0.886,1.056)	0.454
	O_3	0.849(0.792,0.910)	<0.001***	0.910(0.839,0.986)	0.022*
	NO_2	0.869(0.806,0.937)	<0.001***	0.925(0.853,1.003)	0.06
	SO_2	0.962(0.873,1.059)	0.431	0.945(0.855,1.045)	0.273
	CO	1.066(0.986,1.153)	0.108	1.041(0.959,1.129)	0.335
	Cl^-	0.904(0.833,0.981)	0.016*	0.916(0.841,0.996)	0.041*
	$NH4^+$	0.973(0.902,1.050)	0.481	0.943(0.871,1.021)	0.145
	$NO3^-$	0.992(0.921,1.068)	0.825	0.983(0.911,1.061)	0.664
	$SO4^{2-}$	1.085(0.999,1.179)	0.054	1.058(0.972,1.152)	0.192
vascular class (n=1701)	$PM_{2.5}$	1.297(1.224,1.374)	<0.001***	1.279(1.207,1.356)	<0.001***
	PM_1	1.310(1.236,1.388)	<0.001***	1.301(1.227,1.380)	<0.001***
	PM_{10}	1.263(1.185,1.346)	<0.001***	1.247(1.169,1.330)	<0.001***
	O_3	1.125(1.068,1.185)	<0.001***	1.126(1.057,1.200)	<0.001***
	NO_2	1.299(1.224,1.379)	<0.001***	1.297(1.217,1.382)	<0.001***
	SO_2	1.272(1.183,1.367)	<0.001***	1.270(1.181,1.365)	<0.001***
	CO	1.154(1.087,1.224)	<0.001***	1.166(1.097,1.238)	<0.001***
	Cl^-	1.314(1.233,1.399)	<0.001***	1.308(1.226,1.395)	<0.001***
	$NH4^+$	1.270(1.199,1.346)	<0.001***	1.261(1.190,1.336)	<0.001***
	$NO3^-$	1.323(1.250,1.400)	<0.001***	1.304(1.232,1.380)	<0.001***
	$SO4^{2-}$	1.211(1.140,1.287)	<0.001***	1.228(1.155,1.307)	<0.001***

$*P < 0.05$; $**P < 0.01$; $***P < 0.001$

Notes:

Abbreviations: .odds ratio: OR; 95% confidence Interval: 95% CI; IQR, inter-quartile range.

Impact estimates were calculated for each quartile increase in the 3-year average concentration of air pollutants.

Model 1: Model incorporating age, marital status, education state, sex and health status

Model 2: Model 1 + patients' BMI, lifestyle habits (including alcohol consumption, smoking), cooking fuel, place of residence, economic region category, public insurance, depression, physical activity, social activity, and meteorological factors (temperature, relative humidity).

Table S6-1. Results of the interaction analyses(Cross-sectional study).

	Pollutants	age					sex				
class	(IQR)	<60	P-valve	>=60	P-valve	interaction	male	P-valve	female	P-valve	interaction
Respiratory class	PM2.5	1.263 (1.007 ,1.584)	0.044*	1.041 (0.978 ,1.109)	0.206	0.937	1.104 (0.960 ,1.269)	0.166	1.081 (0.988 ,1.181)	0.088	0.981
	PM1	1.220 (0.971 ,1.533)	0.088	1.035 (0.972 ,1.102)	0.282	0.845	1.085 (0.944 ,1.248)	0.251	1.070 (0.980 ,1.169)	0.131	0.940
	PM10	1.408 (1.093 ,1.813)	0.008**	1.048 (0.975 ,1.126)	0.208	0.514	1.160 (0.989 ,1.360)	0.069	1.095 (0.990 ,1.212)	0.079	0.896
	NO2	1.128 (0.884 ,1.440)	0.332	1.005 (0.939 ,1.076)	0.881	0.465	1.024 (0.880 ,1.191)	0.760	1.019 (0.928 ,1.120)	0.689	0.991
	SO2	1.327 (1.007 ,1.749)	0.044*	1.020 (0.940 ,1.106)	0.642	0.115	1.111 (0.926 ,1.332)	0.256	1.056 (0.945 ,1.179)	0.337	0.967
	O3	0.916 (0.731 ,1.148)	0.447	0.979 (0.914 ,1.049)	0.547	0.253	0.906 (0.778 ,1.056)	0.207	0.999 (0.910 ,1.098)	0.988	0.399
	CO	1.442 (1.130 ,1.840)	0.003**	1.022 (0.952 ,1.096)	0.550	0.900	1.158 (0.988 ,1.356)	0.070	1.070 (0.971 ,1.178)	0.172	0.543
	Cl-	1.224 (0.951 ,1.576)	0.117	1.001 (0.933 ,1.075)	0.977	0.295	1.040 (0.889 ,1.217)	0.625	1.036 (0.938 ,1.144)	0.488	0.534
	NH4+	1.328 (1.052 ,1.675)	0.017*	1.041 (0.976 ,1.109)	0.224	0.822	1.167 (1.010 ,1.347)	0.036*	1.059 (0.969 ,1.158)	0.204	0.429
	NO3-	1.200 (0.972 ,1.482)	0.089	1.040 (0.978 ,1.105)	0.215	0.890	1.124 (0.980 ,1.288)	0.094	1.055 (0.969 ,1.148)	0.218	0.596
	SO42-	1.421 (1.102 ,1.833)	0.007**	1.087 (1.012 ,1.167)	0.021*	0.727	1.240 (1.059 ,1.451)	0.007**	1.133 (1.025 ,1.253)	0.015*	0.514
	PM2.5	**0.869 (0.760 ,0.993)**	**0.040***	**1.011 (0.963 ,1.061)**	**0.667**	**0.016***	0.975 (0.867 ,1.097)	0.678	0.980 (0.931 ,1.032)	0.446	0.212
Stomach-arthritis class	PM1	**0.825 (0.724 ,0.940)**	**0.004****	**1.042 (1.007 ,1.059)**	**0.034***	**0.012***	0.918 (0.818 ,1.030)	0.144	0.969 (0.921 ,1.019)	0.223	0.424
	PM10	0.866 (0.745 ,1.006)	0.060	1.013 (0.959 ,1.069)	0.655	0.058	0.935 (0.819 ,1.067)	0.317	0.997 (0.940 ,1.057)	0.908	0.873
	NO2	**0.757 (0.663 ,0.864)**	**<0.000*****	**1.021 (1.009 ,1.066)**	**0.041***	**<0.000*****	0.879 (0.777 ,0.995)	0.041	0.967 (0.917 ,1.021)	0.224	0.797
	SO2	0.888 (0.748 ,1.055)	0.176	0.984 (0.924 ,1.047)	0.608	0.613	0.926 (0.794 ,1.080)	0.328	0.973 (0.911 ,1.040)	0.421	0.874
	O3	**0.839 (0.733 ,0.961)**	**0.011***	**0.965 (0.917 ,1.015)**	**0.168**	**0.018***	0.940 (0.830 ,1.065)	0.335	0.929 (0.880 ,0.980)	0.007**	0.145
	CO	1.004 (0.882 ,1.143)	0.954	1.044 (0.990 ,1.102)	0.114	0.163	1.044 (0.920 ,1.186)	0.504	1.029 (0.975 ,1.086)	0.296	0.441
	Cl-	0.790 (0.689 ,0.906)	0.001**	0.989 (0.936 ,1.045)	0.689	0.071	**0.837 (0.735 ,0.953)**	**0.007**	**1.107 (1.052 ,1.154)**	**0.032****	**0.049***
	NH4+	**0.836 (0.729 ,0.957)**	**0.010***	**1.057 (1.023 ,1.097)**	**0.011***	**0.022***	0.935 (0.829 ,1.054)	0.272	0.969 (0.919 ,1.021)	0.241	0.288
	NO3-	**0.881 (0.771 ,1.008)**	**0.065**	**1.011 (0.964 ,1.059)**	**0.654**	**0.019***	0.952 (0.849 ,1.069)	0.407	0.996 (0.946 ,1.048)	0.877	0.425
	SO42-	**0.961 (0.838 ,1.103)**	**0.571**	**1.064 (1.008 ,1.124)**	**0.025**	**0.015***	**1.101 (1.049 ,1.252)**	**0.034***	**1.018 (0.962 ,1.077)**	**0.538**	**0.048***
Vascular class	PM2.5	1.296 (1.174 ,1.430)	<0.001***	1.125 (1.084 ,1.167)	<0.001***	0.804	1.278 (1.172 ,1.394)	<0.001***	1.127 (1.082 ,1.174)	<0.001***	0.559

PM1	1.331 (1.205 ,1.469)	<0.001***	1.132 (1.090 ,1.175)	<0.001***	0.648	1.293 (1.185 ,1.410)	<0.001***	1.139 (1.093 ,1.187)	<0.001***	0.723
PM10	1.220 (1.095 ,1.358)	<0.001***	1.114 (1.068 ,1.161)	<0.001***	0.732	1.238 (1.125 ,1.363)	<0.001***	1.108 (1.059 ,1.160)	<0.001***	0.677
NO2	1.283 (1.158 ,1.421)	<0.001***	1.154 (1.107 ,1.204)	<0.001***	0.326	1.273 (1.158 ,1.398)	<0.001***	1.151 (1.101 ,1.204)	<0.001***	0.724
SO2	1.227 (1.089 ,1.382)	0.001**	1.141 (1.088 ,1.195)	<0.001***	0.614	1.254 (1.126 ,1.397)	<0.001***	1.129 (1.073 ,1.187)	<0.001***	0.940
O3	1.175 (1.057 ,1.307)	0.003**	1.049 (1.007 ,1.092)	0.021*	0.890	1.113 (1.012 ,1.223)	0.027*	1.067 (1.021 ,1.116)	0.004**	0.413
CO	1.144 (1.038 ,1.260)	0.007**	1.088 (1.045 ,1.134)	<0.001***	0.665	1.165 (1.064 ,1.276)	0.001**	1.080 (1.035 ,1.127)	<0.001***	0.911
Cl-	1.318 (1.189 ,1.462)	<0.001***	1.138 (1.090 ,1.188)	<0.001***	0.813	1.296 (1.178 ,1.426)	<0.001***	1.140 (1.089 ,1.192)	<0.001***	0.939
NH4+	1.284 (1.166 ,1.414)	<0.001***	1.116 (1.075 ,1.158)	<0.001***	0.670	1.241 (1.139 ,1.352)	<0.001***	1.128 (1.083 ,1.175)	<0.001***	0.908
NO3-	1.331 (1.208 ,1.466)	<0.001***	1.135 (1.094 ,1.177)	<0.001***	0.786	1.287 (1.183 ,1.400)	<0.001***	1.145 (1.100 ,1.191)	<0.001***	0.753
SO42-	1.187 (1.075 ,1.312)	0.001**	1.111 (1.067 ,1.157)	<0.001***	0.717	1.254 (1.144 ,1.376)	<0.001***	1.090 (1.044 ,1.138)	<0.001***	0.278

Table S6-2. Results of the interaction analyses.(Cross-sectional study)

	Pollutants	health						
class	(IQR)	0	P-value	1	P-value	2	P-value	interaction
Respiratory class	PM2.5	1.134 (0.831 ,1.547)	0.427	1.034 (0.957 ,1.117)	0.393	1.061 (0.999 ,1.127)	0.053	0.973
	PM1	1.072 (0.787 ,1.459)	0.659	1.039 (0.962 ,1.123)	0.331	1.048 (0.988 ,1.112)	0.121	0.871
	PM10	1.187 (0.840 ,1.678)	0.332	1.053 (0.964 ,1.151)	0.252	1.074 (1.004 ,1.150)	0.039*	0.933
	NO2	0.989 (0.708 ,1.381)	0.949	0.977 (0.900 ,1.060)	0.570	1.058 (0.990 ,1.130)	0.096	0.657
	SO2	1.358 (0.907 ,2.035)	0.137	0.991 (0.898 ,1.092)	0.849	1.076 (0.995 ,1.164)	0.066	0.800
	O3	0.966 (0.697 ,1.337)	0.834	0.921 (0.845 ,1.004)	0.062	1.017 (0.953 ,1.084)	0.616	0.791
	CO	0.980 (0.702 ,1.369)	0.905	1.081 (0.990 ,1.180)	0.081	1.053 (0.985 ,1.126)	0.128	0.857
	Cl-	1.129 (0.793 ,1.607)	0.501	0.977 (0.898 ,1.064)	0.598	1.061 (0.991 ,1.135)	0.091	0.729
	NH4+	1.111 (0.814 ,1.517)	0.507	1.062 (0.982 ,1.149)	0.134	1.049 (0.987 ,1.115)	0.122	0.690
	NO3-	1.160 (0.856 ,1.571)	0.339	1.044 (0.969 ,1.125)	0.257	1.039 (0.980 ,1.101)	0.200	0.482
	SO42-	1.287 (0.910 ,1.821)	0.154	1.124 (1.028 ,1.228)	0.010**	1.059 (0.990 ,1.132)	0.093	0.414

	PM2.5	0.797 (0.648 ,0.981)	0.032*	0.974 (0.919 ,1.033)	0.379	1.012 (0.972 ,1.054)	0.567	0.123
Stomach-arthritis class	PM1	0.755 (0.616 ,0.925)	0.007**	0.957 (0.904 ,1.014)	0.134	0.997 (0.958 ,1.038)	0.890	0.144
	PM10	0.790 (0.623 ,1.002)	0.052	0.988 (0.925 ,1.056)	0.722	1.001 (0.956 ,1.049)	0.956	0.459
	NO2	**0.675 (0.544 ,0.837)**	<0.001***	**0.946 (0.891 ,1.005)**	**0.072**	**1.004 (0.961 ,1.048)**	**0.856**	0.021*
	SO2	0.775 (0.584 ,1.027)	0.076	0.969 (0.899 ,1.044)	0.405	0.996 (0.944 ,1.051)	0.894	0.956
	O3	0.906 (0.726 ,1.132)	0.386	0.920 (0.867 ,0.977)	0.007**	0.982 (0.940 ,1.026)	0.428	0.354
	CO	0.919 (0.730 ,1.155)	0.468	1.033 (0.973 ,1.096)	0.284	1.022 (0.978 ,1.069)	0.333	0.605
	Cl-	0.693 (0.549 ,0.876)	0.002**	0.962 (0.903 ,1.024)	0.224	0.982 (0.939 ,1.027)	0.430	0.375
	NH4+	0.774 (0.625 ,0.959)	0.019*	0.974 (0.918 ,1.034)	0.383	0.987 (0.946 ,1.029)	0.527	0.498
	NO3-	0.824 (0.670 ,1.013)	0.066	0.985 (0.930 ,1.043)	0.606	1.003 (0.964 ,1.044)	0.870	0.377
	SO42-	0.843 (0.679 ,1.047)	0.123	1.049 (0.984 ,1.118)	0.141	1.027 (0.981 ,1.074)	0.259	0.381
	PM2.5	1.376 (1.187 ,1.594)	<0.001***	1.103 (1.061 ,1.148)	<0.001***	1.095 (1.055 ,1.138)	<0.001***	0.737
Vascular class	PM1	1.450 (1.244 ,1.689)	<0.001***	1.110 (1.067 ,1.155)	<0.001***	1.099 (1.059 ,1.142)	<0.001***	0.568
	PM10	1.279 (1.092 ,1.499)	0.002**	1.089 (1.042 ,1.138)	<0.001***	1.085 (1.040 ,1.133)	<0.001***	0.879
	NO2	1.447 (1.220 ,1.717)	<0.001***	1.112 (1.065 ,1.161)	<0.001***	1.114 (1.070 ,1.161)	<0.001***	0.581
	SO2	1.374 (1.150 ,1.642)	<0.001***	1.094 (1.042 ,1.149)	<0.001***	1.105 (1.053 ,1.160)	<0.001***	0.803
	O3	1.217 (1.044 ,1.419)	0.012*	1.052 (1.007 ,1.099)	0.024*	1.035 (0.994 ,1.079)	0.095	0.058
	CO	1.224 (1.055 ,1.420)	0.008**	1.067 (1.023 ,1.112)	0.003**	1.059 (1.017 ,1.103)	0.005**	0.777
	Cl-	1.348 (1.149 ,1.581)	<0.001***	1.110 (1.062 ,1.160)	<0.001***	1.120 (1.074 ,1.167)	<0.001***	0.446
	NH4+	1.366 (1.181 ,1.579)	<0.001***	1.096 (1.054 ,1.140)	<0.001***	1.087 (1.046 ,1.129)	<0.001***	0.609
	NO3-	1.460 (1.258 ,1.694)	<0.001***	1.115 (1.073 ,1.159)	<0.001***	1.094 (1.054 ,1.135)	<0.001***	0.241
	SO42-	1.316 (1.132 ,1.530)	<0.001***	1.078 (1.034 ,1.124)	<0.001***	1.074 (1.030 ,1.119)	0.001**	0.854

Table S6-3. Results of the interaction analyses.(Cross-sectional study)

	Pollutants	drink					smoke				
class	(IQR)	0	P-value	1	P-value	interaction	0	P-value	1	P-value	interaction
Respiratory class	PM2.5	1.091 (0.935 ,1.274)	0.267	1.067 (0.988 ,1.154)	0.100	0.488	1.127 (0.941 ,1.351)	0.195	1.053 (0.983 ,1.127)	0.143	0.744
	PM1	1.060 (0.909 ,1.235)	0.459	1.066 (0.986 ,1.152)	0.107	0.341	1.089 (0.910 ,1.304)	0.351	1.046 (0.977 ,1.120)	0.195	0.582
	PM10	1.116 (0.938 ,1.327)	0.218	1.092 (0.998 ,1.194)	0.055	0.386	1.171 (0.954 ,1.437)	0.131	1.073 (0.992 ,1.161)	0.080	0.745
	NO2	0.997 (0.843 ,1.179)	0.971	1.031 (0.949 ,1.120)	0.474	0.346	0.934 (0.774 ,1.127)	0.473	1.041 (0.965 ,1.123)	0.295	0.119
	SO2	1.006 (0.826 ,1.224)	0.953	1.097 (0.993 ,1.213)	0.070	0.169	1.079 (0.860 ,1.353)	0.510	1.054 (0.964 ,1.152)	0.249	0.565
	O3	0.993 (0.843 ,1.170)	0.937	0.941 (0.865 ,1.025)	0.164	0.657	1.017 (0.831 ,1.244)	0.870	0.950 (0.884 ,1.022)	0.169	0.656
	CO	1.213 (1.017 ,1.447)	0.032*	1.036 (0.952 ,1.128)	0.406	0.937	1.088 (0.895 ,1.322)	0.398	1.081 (0.999 ,1.169)	0.052	0.326
	Cl-	1.017 (0.853 ,1.213)	0.847	1.039 (0.954 ,1.131)	0.383	0.528	1.023 (0.838 ,1.248)	0.822	1.023 (0.946 ,1.105)	0.572	0.755
	NH4+	1.077 (0.923 ,1.257)	0.349	1.092 (1.008 ,1.183)	0.032*	0.246	1.064 (0.888 ,1.276)	0.501	1.079 (1.006 ,1.158)	0.034*	0.234
	NO3-	1.086 (0.936 ,1.259)	0.277	1.060 (0.984 ,1.143)	0.127	0.559	1.077 (0.907 ,1.280)	0.398	1.057 (0.988 ,1.129)	0.105	0.461
	SO42-	1.271 (1.066 ,1.516)	0.007**	1.093 (1.001 ,1.192)	0.046*	0.719	1.148 (0.936 ,1.410)	0.186	1.139 (1.054 ,1.230)	<0.001***	0.142
Stomach-arthritis class	PM2.5	0.940 (0.845 ,1.045)	0.250	0.990 (0.936 ,1.048)	0.731	0.463	0.981 (0.885 ,1.087)	0.711	0.967 (0.912 ,1.026)	0.264	0.510
	PM1	0.903 (0.814 ,1.002)	0.055	0.970 (0.918 ,1.026)	0.292	0.466	0.953 (0.861 ,1.055)	0.354	0.942 (0.890 ,0.998)	0.042*	0.348
	PM10	0.944 (0.838 ,1.064)	0.346	0.985 (0.924 ,1.051)	0.649	0.591	0.990 (0.882 ,1.112)	0.865	0.958 (0.896 ,1.024)	0.208	0.378
	NO2	0.857 (0.768 ,0.958)	0.006**	0.979 (0.922 ,1.039)	0.486	0.135	0.934 (0.840 ,1.039)	0.208	0.934 (0.878 ,0.994)	0.033*	0.734
	SO2	0.925 (0.806 ,1.061)	0.266	0.970 (0.901 ,1.044)	0.415	0.779	0.954 (0.836 ,1.090)	0.492	0.952 (0.882 ,1.028)	0.207	0.344
	O3	0.866 (0.775 ,0.967)	0.011*	0.962 (0.907 ,1.021)	0.204	0.235	0.882 (0.789 ,0.985)	0.026	0.954 (0.899 ,1.013)	0.124	0.311
	CO	1.028 (0.919 ,1.150)	0.628	1.032 (0.972 ,1.095)	0.310	0.552	1.071 (0.961 ,1.192)	0.214	1.008 (0.946 ,1.073)	0.811	0.356
	Cl-	0.878 (0.781 ,0.986)	0.029*	0.957 (0.900 ,1.018)	0.163	0.715	0.963 (0.861 ,1.076)	0.504	0.902 (0.845 ,0.963)	0.002**	0.067
	NH4+	0.926 (0.832 ,1.031)	0.162	0.965 (0.911 ,1.023)	0.236	0.757	0.950 (0.855 ,1.056)	0.341	0.951 (0.896 ,1.010)	0.100	0.480
	NO3-	0.978 (0.881 ,1.086)	0.683	0.976 (0.923 ,1.031)	0.384	0.993	0.990 (0.895 ,1.095)	0.845	0.968 (0.913 ,1.025)	0.264	0.511
	SO42-	1.015 (0.906 ,1.136)	0.800	1.048 (0.983 ,1.117)	0.150	0.267	1.035 (0.924 ,1.159)	0.549	1.038 (0.974 ,1.106)	0.254	0.853
Vascular class	**PM2.5**	**1.188 (1.099 ,1.284)**	**<0.001*** **	**1.173 (1.121 ,1.228)**	**<0.001*** **	**0.034***	1.263 (1.169 ,1.364)	<0.001***	1.127 (1.077 ,1.180)	<0.001***	0.676

PM1	1.219 (1.127 ,1.319)	<0.001***	1.177 (1.125 ,1.232)	<0.001***	0.076	1.310 (1.211 ,1.417)	<0.001***	1.122 (1.072 ,1.174)	<0.001***	0.268
PM10	1.151 (1.055 ,1.255)	0.002**	1.151 (1.095 ,1.210)	<0.001***	0.140	1.204 (1.106 ,1.312)	<0.001***	1.119 (1.063 ,1.177)	<0.001***	0.870
NO2	1.261 (1.157 ,1.376)	<0.001***	1.158 (1.103 ,1.216)	<0.001***	0.273	1.337 (1.228 ,1.457)	<0.001***	1.118 (1.064 ,1.175)	<0.001***	0.388
SO2	1.212 (1.098 ,1.338)	<0.001***	1.153 (1.092 ,1.219)	<0.001***	0.536	1.291 (1.172 ,1.421)	<0.001***	1.111 (1.049 ,1.176)	<0.001***	0.346
O3	1.101 (1.011 ,1.200)	0.027*	1.070 (1.019 ,1.123)	0.006**	0.372	1.133 (1.039 ,1.236)	0.005**	1.053 (1.004 ,1.105)	0.035*	0.715
CO	1.143 (1.051 ,1.242)	0.002**	1.090 (1.041 ,1.142)	<0.001***	0.676	1.170 (1.078 ,1.270)	<0.001***	1.076 (1.025 ,1.128)	0.003**	0.337
Cl-	1.245 (1.141 ,1.360)	<0.001***	1.163 (1.108 ,1.222)	<0.001***	0.361	1.304 (1.197 ,1.422)	<0.001***	1.131 (1.075 ,1.190)	<0.001***	0.838
NH4+	1.193 (1.103 ,1.290)	<0.001***	1.153 (1.102 ,1.206)	<0.001***	0.116	1.271 (1.175 ,1.374)	<0.001***	1.105 (1.057 ,1.156)	<0.001***	0.275
NO3-	1.237 (1.146 ,1.335)	<0.001***	1.170 (1.120 ,1.222)	<0.001***	0.120	1.306 (1.211 ,1.409)	<0.001***	1.128 (1.079 ,1.179)	<0.001***	0.393
SO42-	1.136 (1.046 ,1.233)	0.002**	1.148 (1.093 ,1.205)	<0.001***	0.068	1.180 (1.085 ,1.282)	<0.001***	1.117 (1.065 ,1.172)	<0.001***	0.995

*P < 0.05; **P < 0.01; ***P < 0.001

Notes: Abbreviations:IQR, inter-quartile range.

Adjusted:age, sex, meteorological factors (temperature, relative humidity), marital status, education state, health status, patients' BMI, place of residence, social activity, public insurance, lifestyle habits (including alcohol consumption, smoking), cooking fuel, economic region category, depression, physical activity.

Impact estimates have been calculated for each quartile increment in the 3-year mean concentration of air contaminants.

Table S7-1. Results of the interaction analyses.(Longitudinal study)

pollutants	age					sex				
	<60	*P-value*	>=60	*P-value*	*interaction*	male	*P-value*	female	*P-value*	*interaction*
$PM_{2.5}$	1.182(0.975,1.433)	0.089	1.102(0.992,1.225)	0.069	0.614	1.315(1.047,1.651)	0.018*	1.049(0.955,1.151)	0.317	0.462
PM_1	1.295(0.927,1.809)	0.130	1.195(0.993,1.439)	0.059	0.636	1.651(1.099,2.480)	0.016*	1.083(0.921,1.273)	0.336	0.422
PM_{10}	1.121(0.993,1.266)	0.064	1.056(0.992,1.124)	0.090	0.630	1.184(1.031,1.359)	0.016*	1.030(0.972,1.091)	0.318	0.411
NO_2	1.348(0.968,1.876)	0.077	1.207(1.027,1.419)	0.023*	0.622	1.776(1.228,2.568)	0.002**	1.109(0.949,1.296)	0.193	0.465
SO_2	2.743(1.743,4.317)	<0.000***	2.053(1.558,2.707)	<0.000***	0.887	4.292(2.387,7.716)	<0.000***	1.670(1.344,2.075)	<0.000***	0.527
O_3	0.803(0.643,1.003)	0.053	0.885(0.795,0.986)	0.026*	0.584	0.782(0.598,1.022)	0.072	0.885(0.805,0.973)	0.011*	0.976
CO	1.258(1.078,1.467)	0.003**	1.128(1.048,1.214)	0.001**	0.513	1.298(1.113,1.515)	0.001**	1.113(1.035,1.197)	0.004**	0.918
Cl^-	1.274(0.850,1.907)	0.241	1.266(0.952,1.684)	0.105	0.671	1.739(1.017,2.974)	0.043*	1.054(0.856,1.298)	0.620	0.455
NH_4^+	1.121(0.970,1.295)	0.123	1.049(0.974,1.130)	0.206	0.509	1.151(0.980,1.352)	0.087	1.028(0.959,1.102)	0.436	0.725
NO_3^-	1.039(0.963,1.121)	0.329	1.013(0.977,1.051)	0.477	0.496	1.062(0.978,1.154)	0.154	1.003(0.969,1.038)	0.873	0.567
SO_4^{2-}	1.105(0.978,1.249)	0.109	1.052(0.990,1.118)	0.100	0.560	1.127(0.995,1.278)	0.061	1.033(0.974,1.095)	0.283	0.905

Table S7-2. Results of the interaction analyses.(Longitudinal study)

pollutants	health						
	0	P-value	1	P-value	2	P-value	interaction
PM2.5	1.203(0.952,1.521)	0.122	1.065(0.959,1.181)	0.239	1.152(1.004,1.323)	0.044*	0.796
PM1	1.382(0.919,2.078)	0.120	1.110(0.925,1.332)	0.261	1.302(1.018,1.665)	0.036*	0.810
PM10	1.133(0.977,1.313)	0.099	1.038(0.973,1.107)	0.257	1.081(0.997,1.171)	0.059	0.991
NO2	1.486(0.997,2.214)	0.052	1.153(0.974,1.366)	0.099	1.276(1.011,1.610)	0.040*	0.853
SO2	3.034(1.820,5.057)	<0.000***	2.170(1.602,2.938)	<0.000***	1.453(0.986,2.143)	0.059	0.385
O3	0.832(0.647,1.070)	0.151	0.874(0.782,0.978)	0.019*	1.033(0.847,1.260)	0.747	0.516

CO	1.331(1.102,1.607)	0.003**	1.120(1.033,1.214)	0.006**	1.102(1.000,1.215)	0.049*	0.940
Cl-	1.510(0.891,2.558)	0.126	1.078(0.845,1.376)	0.545	1.339(0.986,1.818)	0.061	0.730
NH4+	1.107(0.938,1.307)	0.230	1.032(0.957,1.113)	0.419	1.113(0.995,1.244)	0.060	0.672
NO3-	1.037(0.951,1.130)	0.414	1.008(0.971,1.046)	0.691	1.056(0.995,1.119)	0.071	0.785
SO42-	1.112(0.957,1.292)	0.166	1.027(0.967,1.091)	0.388	1.090(0.996,1.194)	0.062	0.604

Table S7-3. Results of the interaction analyses.(Longitudinal study)

pollutants	drink					smoke				
	0	*P-value*	1	*P-value*	*interaction*	0	*P-value*	1	*P-value*	*interaction*
$PM_{2.5}$	1.166(0.970,1.402)	0.101	1.119(1.005,1.246)	0.040*	0.662	1.107(0.928,1.321)	0.260	1.155(1.028,1.297)	0.015*	0.345
PM_1	1.292(0.933,1.790)	0.123	1.235(1.023,1.491)	0.028*	0.575	1.203(0.884,1.637)	0.241	1.289(1.045,1.590)	0.018*	0.365
PM_{10}	1.092(0.977,1.220)	0.123	1.077(1.007,1.152)	0.030*	0.457	1.068(0.958,1.191)	0.235	1.090(1.014,1.171)	0.019*	0.335
NO_2	1.346(0.986,1.838)	0.061	1.261(1.059,1.502)	0.009**	0.703	1.251(0.929,1.684)	0.140	1.348(1.113,1.632)	0.002**	0.306
SO_2	2.936(1.893,4.556)	<0.000***	1.949(1.449,2.621)	<0.000***	0.258	2.945(1.952,4.443)	<0.000***	2.105(1.528,2.901)	<0.000***	0.601
O_3	0.789(0.644,0.965)	0.021*	0.913(0.805,1.036)	0.160	0.784	0.789(0.660,0.942)	0.009**	0.882(0.757,1.026)	0.104	0.951
CO	1.252(1.083,1.447)	0.002**	1.130(1.047,1.220)	0.002**	0.769	1.263(1.102,1.448)	0.001**	1.133(1.044,1.231)	0.003**	0.955
Cl^-	1.262(0.823,1.936)	0.287	1.252(0.984,1.592)	0.067	0.302	1.142(0.774,1.687)	0.504	1.357(1.029,1.790)	0.031*	0.360
NH_4^+	1.109(0.970,1.267)	0.130	1.058(0.979,1.144)	0.153	0.977	1.058(0.928,1.206)	0.397	1.083(0.998,1.175)	0.055	0.518
NO_3^-	1.032(0.964,1.105)	0.364	1.026(0.986,1.068)	0.209	0.819	1.005(0.941,1.073)	0.887	1.038(0.995,1.082)	0.087	0.334
SO_4^{2-}	1.115(0.994,1.252)	0.064	1.050(0.986,1.119)	0.130	0.680	1.081(0.969,1.207)	0.163	1.061(0.993,1.133)	0.081	0.876

*P < 0.05; **P < 0.01; ***P < 0.001

Notes: Adjusted: age, sex, meteorological factors (temperature, relative humidity), marital status, education state, health status, patients' BMI, place of residence, social activity, public insurance, lifestyle habits (including alcohol consumption, smoking), cooking fuel, economic region category, depression, physical activity.

Air pollutants $PM_{2.5}$, PM_1, PM_{10}, NO_2, SO_2 (increasing by 10 μg/m3 each), CO (increasing by 0.1 mg/m3), and PMC (Cl^-, NH_4^+, NO_3^-, SO_4^{2-}) (increasing by 1 μg/m3 each) are used to calculate the impact estimate.

Impact estimates have been calculated for each quartile increment in the 3-year mean concentration of air contaminants.

Table S8-1. Results of the stratified analyses.(Cross-sectional study)

	Pollutants	age				<60 vs >=60	sex				Male vs female
class	(IQR)	<60	*P-valve*	>=60	*P-valve*	*Fisher's permutation test*	male	*P-valve*	female	*P-valve*	*Fisher's permutation test*
Respiratory class	$PM_{2.5}$	1.242(0.993,1.553)	0.068	1.086(0.959,1.231)	0.154	0.090	1.101(0.958,1.265)	0.163	1.164(0.976,1.387)	0.12	0.250
	PM_1	1.199(0.958,1.500)	0.131	1.072(0.947,1.214)	0.220	0.200	1.082(0.942,1.243)	0.248	1.144(0.962,1.361)	0.172	0.370
	PM_{10}	**1.389(1.082,1.783)**	**0.011***	**1.091(0.946,1.260)**	**0.207**	**<0.001*** **	1.155(0.985,1.354)	0.079	1.181(0.967,1.442)	0.126	0.390
	NO_2	1.081(0.852,1.372)	0.574	1.019(0.890,1.165)	0.748	0.210	1.020(0.877,1.186)	0.732	1.038(0.862,1.250)	0.781	0.440
	SO_2	**1.318(1.002,1.734)**	**0.041***	**1.047(0.891,1.232)**	**0.562**	**0.050***	1.113(0.929,1.334)	0.234	1.121(0.901,1.394)	0.366	0.370
	O_3	0.911(0.729,1.138)	0.403	0.971(0.846,1.114)	0.711	0.370	0.904(0.776,1.052)	0.222	1.022(0.849,1.231)	0.871	0.160
	CO	**1.433(1.128,1.821)**	**0.004****	**1.040(0.904,1.196)**	**0.652**	**0.020***	1.158(0.989,1.356)	0.078	1.121(0.928,1.355)	0.288	0.390
	Cl^-	1.180(0.922,1.509)	0.222	1.010(0.877,1.162)	0.787	0.080	1.042(0.891,1.219)	0.558	1.060(0.872,1.288)	0.636	0.490
	NH_4^+	**1.298(1.033,1.633)**	**0.03***	**1.087(0.957,1.234)**	**0.130**	**0.030***	1.167(1.012,1.347)	0.032*	1.113(0.935,1.326)	0.267	0.460
	NO_3^-	1.187(0.963,1.463)	0.127	1.078(0.955,1.218)	0.150	0.140	1.120(0.978,1.283)	0.093	1.110(0.939,1.312)	0.267	0.320
	**SO_4^{2-}**	**1.412(1.097,1.816)**	**0.009****	**1.170(1.016,1.346)**	**0.019***	**0.020***	1.239(1.059,1.449)	0.007**	1.248(1.024,1.521)	0.031*	0.380
Stomach-arthritis class	$PM_{2.5}$	**0.869(0.760,0.993)**	**0.034***	**1.033(0.938,1.138)**	**0.499**	**0.010****	0.984(0.874,1.108)	0.703	0.966(0.871,1.072)	0.489	0.430
	PM_1	**0.829(0.728,0.944)**	**0.004****	**0.998(0.907,1.099)**	**0.967**	**<0.001*** **	0.926(0.825,1.040)	0.166	0.945(0.854,1.046)	0.255	0.400
	PM_{10}	**0.859(0.739,0.999)**	**0.056**	**1.029(0.922,1.149)**	**0.592**	**0.010****	0.941(0.823,1.076)	0.314	0.988(0.878,1.111)	0.855	0.190
	NO_2	**0.761(0.667,0.869)**	**<0.001*** **	**1.047(0.942,1.164)**	**0.503**	**<0.001*** **	0.892(0.788,1.010)	0.057	0.953(0.856,1.062)	0.293	0.250
	SO_2	0.892(0.751,1.060)	0.141	0.981(0.865,1.112)	0.765	0.170	0.931(0.798,1.087)	0.326	0.956(0.837,1.091)	0.458	0.250
	O_3	0.845(0.738,0.967)	0.012*	0.946(0.854,1.048)	0.223	0.070	0.950(0.838,1.077)	0.346	0.881(0.791,0.980)	0.012*	0.090
	CO	1.002(0.880,1.141)	0.96	1.079(0.970,1.201)	0.178	0.180	1.024(0.902,1.163)	0.733	1.050(0.943,1.168)	0.374	0.360
	Cl^-	**0.791(0.689,0.907)**	**<0.001*** **	**1.000(0.895,1.117)**	**0.987**	**<0.001*** **	**0.856(0.751,0.975)**	**0.014***	**1.090(1.012,1.161)**	**0.018***	**0.040***
	NH_4^+	**0.832(0.726,0.953)**	**0.008****	**1.004(0.909,1.109)**	**0.850**	**<0.001*** **	0.950(0.842,1.073)	0.374	0.936(0.842,1.040)	0.275	0.500
	NO_3^-	**0.889(0.777,1.016)**	**0.077**	**1.031(0.938,1.133)**	**0.493**	**0.040***	0.967(0.861,1.086)	0.506	0.996(0.900,1.104)	0.954	0.340
	SO_4^{2-}	**0.956(0.832,1.099)**	**0.599**	**1.130(1.013,1.260)**	**0.015***	**0.049***	1.096(0.963,1.249)	0.173	1.030(0.920,1.154)	0.449	0.340
Vascular class	$PM_{2.5}$	**1.310(1.189,1.443)**	**<0.001*** **	**1.258(1.168,1.354)**	**<0.001*** **	**0.040***	1.285(1.180,1.400)	<0.001***	1.262(1.165,1.368)	<0.001***	0.450

PM_1	**1.344(1.220,1.481)**	**<0.001*** **	**1.275(1.183,1.373)**	**<0.001*** **	**0.040***	1.300(1.193,1.417)	<0.001***	1.291(1.191,1.399)	<0.001***	0.410
PM_{10}	1.252(1.126,1.392)	<0.001***	1.237(1.139,1.343)	<0.001***	0.250	1.255(1.142,1.380)	<0.001***	1.227(1.123,1.342)	<0.001***	0.420
NO_2	1.285(1.162,1.422)	<0.001***	1.318(1.212,1.433)	<0.001***	0.380	1.269(1.156,1.393)	<0.001***	1.311(1.201,1.432)	<0.001***	0.450
SO_2	1.237(1.101,1.391)	<0.001***	1.288(1.174,1.414)	<0.001***	0.430	1.264(1.136,1.407)	<0.001***	1.255(1.137,1.387)	<0.001***	0.400
O_3	1.168(1.052,1.296)	0.001**	1.103(1.018,1.195)	0.017*	0.090	1.116(1.017,1.225)	0.019*	1.131(1.036,1.234)	0.004**	0.320
CO	1.138(1.036,1.250)	0.006**	1.182(1.090,1.282)	<0.001***	0.420	1.172(1.072,1.281)	<0.001***	1.152(1.060,1.252)	0.001**	0.420
Cl^-	1.341(1.212,1.484)	<0.001***	1.286(1.181,1.399)	<0.001***	0.140	1.305(1.187,1.435)	<0.001***	1.296(1.186,1.417)	<0.001***	0.420
NH_4^+	1.298(1.181,1.427)	<0.001***	1.233(1.145,1.328)	<0.001***	0.070	1.250(1.149,1.361)	<0.001***	1.255(1.158,1.360)	<0.001***	0.320
NO_3^-	1.351(1.228,1.487)	<0.001***	1.274(1.186,1.369)	<0.001***	0.080	1.296(1.192,1.408)	<0.001***	1.298(1.200,1.404)	<0.001***	0.400
SO_4^{2-}	1.201(1.089,1.325)	<0.001***	1.230(1.135,1.333)	<0.001***	0.360	1.265(1.155,1.386)	<0.001***	1.186(1.089,1.292)	<0.001***	0.270

Table S8-2. Results of the stratified analyses.(Cross-sectional study)

class	Pollutants (IQR)	health 0	*P-value*	1	*P-value*	2	*P-value*	*0 vs 1* *Fisher's permutation test*	*0 vs 2* *Fisher's permutation test*	*1 vs 2* *Fisher's permutation test*
Respiratory class	$PM_{2.5}$	1.145(0.837,1.565)	0.371	1.073(0.920,1.250)	0.402	1.212(1.013,1.450)	0.043*	0.290	0.400	0.310
	PM_1	1.090(0.800,1.484)	0.565	1.081(0.926,1.261)	0.342	1.168(0.978,1.394)	0.124	0.490	0.460	0.440
	PM_{10}	1.205(0.849,1.712)	0.287	1.104(0.926,1.316)	0.303	1.259(1.027,1.544)	0.041*	0.260	0.430	0.360
	NO_2	0.988(0.706,1.382)	0.956	0.953(0.809,1.123)	0.589	1.190(0.979,1.447)	0.120	0.490	0.320	0.090
	SO_2	1.342(0.895,2.010)	0.133	0.984(0.810,1.194)	0.821	1.276(1.010,1.612)	0.043*	0.070	0.350	0.090
	O_3	1.024(0.736,1.424)	0.956	**0.854(0.719,1.014)**	**0.073**	**1.058(0.873,1.281)**	**0.635**	**0.080**	**0.400**	**0.040***
	CO	0.954(0.685,1.329)	0.897	1.161(0.977,1.380)	0.103	1.180(0.968,1.439)	0.137	0.170	0.190	0.390
	Cl^-	1.137(0.796,1.624)	0.467	0.958(0.809,1.135)	0.593	1.211(0.989,1.483)	0.088	0.140	0.480	0.140
	NH_4^+	1.109(0.810,1.519)	0.465	1.144(0.978,1.339)	0.103	1.170(0.975,1.405)	0.114	0.480	0.470	0.410
	NO_3^-	1.179(0.867,1.604)	0.295	1.098(0.947,1.275)	0.229	1.131(0.951,1.346)	0.204	0.350	0.280	0.440
	SO_4^{2-}	1.262(0.891,1.789)	0.154	1.264(1.061,1.506)	0.011*	1.203(0.984,1.469)	0.095	0.350	0.200	0.310

	$PM_{2.5}$	**0.806(0.654,0.992)**	**0.040***	0.964(0.857,1.085)	0.490	**1.046(0.925,1.183)**	**0.549**	0.080	0.050*	0.330
Stomach-arthritis class	PM_1	**0.769(0.627,0.942)**	**0.010***	0.931(0.830,1.045)	0.193	**1.000(0.887,1.129)**	**0.927**	0.070	**0.030***	0.480
	PM_{10}	0.802(0.632,1.019)	0.076	0.983(0.860,1.124)	0.751	1.009(0.877,1.161)	0.958	0.100	0.110	0.360
	NO_2	**0.671(0.540,0.833)**	**<0.001*****	**0.922(0.817,1.041)**	**0.151**	**1.029(0.904,1.173)**	**0.790**	**<0.001*****	**<0.001*****	0.310
	SO_2	0.774(0.583,1.028)	0.084	0.958(0.825,1.113)	0.497	0.996(0.847,1.170)	0.861	0.080	0.150	0.470
	O_3	0.933(0.746,1.167)	0.508	0.859(0.763,0.969)	0.012*	0.958(0.839,1.093)	0.415	0.190	0.410	0.220
	CO	0.910(0.724,1.143)	0.397	1.051(0.934,1.183)	0.417	1.078(0.943,1.232)	0.326	0.180	0.140	0.430
	Cl^-	**0.700(0.553,0.885)**	**0.003****	**0.951(0.838,1.078)**	**0.425**	**0.957(0.836,1.097)**	**0.469**	**0.020***	**<0.001*****	0.190
	NH_4^+	**0.780(0.628,0.967)**	**0.021***	**0.964(0.855,1.087)**	**0.586**	0.966(0.851,1.097)	0.594	**0.010****	0.090	0.320
	NO_3^-	0.833(0.677,1.026)	0.078	0.994(0.885,1.116)	0.883	1.014(0.898,1.145)	0.847	0.100	0.070	0.460
	SO_4^{2-}	**0.840(0.676,1.045)**	**0.138**	**1.107(0.974,1.259)**	**0.099**	1.088(0.948,1.249)	0.211	**<0.001*****	0.050	0.200
	$PM_{2.5}$	1.371(1.185,1.586)	<0.001***	1.228(1.135,1.329)	<0.001***	1.329(1.187,1.487)	<0.001***	0.130	0.280	0.240
Vascular class	PM_1	**1.452(1.249,1.688)**	**<0.001*****	**1.240(1.146,1.342)**	**<0.001*****	1.343(1.201,1.502)	<0.001***	**0.030***	0.190	0.210
	PM_{10}	1.299(1.111,1.520)	0.001**	1.204(1.103,1.313)	<0.001***	1.303(1.149,1.478)	<0.001***	0.160	0.430	0.220
	NO_2	1.391(1.177,1.644)	<0.001***	1.243(1.141,1.354)	<0.001***	1.377(1.220,1.554)	<0.001***	0.110	0.330	0.170
	SO_2	1.366(1.146,1.628)	0.001**	1.202(1.091,1.325)	<0.001***	1.345(1.165,1.553)	<0.001***	0.090	0.480	0.200
	O_3	1.196(1.027,1.392)	0.014*	1.119(1.026,1.222)	0.01*	1.107(0.981,1.249)	0.076	0.190	0.220	0.270
	CO	1.214(1.050,1.405)	0.011*	1.133(1.043,1.230)	0.002**	1.210(1.074,1.363)	0.002**	0.160	0.340	0.320
	Cl^-	1.345(1.148,1.576)	<0.001***	1.251(1.146,1.366)	<0.001***	1.402(1.238,1.587)	<0.001***	0.210	0.430	0.170
	NH_4^+	1.352(1.172,1.559)	<0.001***	1.211(1.120,1.309)	<0.001***	1.292(1.153,1.447)	<0.001***	0.080	0.310	0.150
	NO_3^-	**1.441(1.245,1.669)**	**<0.001*****	**1.255(1.163,1.354)**	**<0.001*****	1.318(1.180,1.472)	<0.001***	**<0.001*****	0.160	0.250
	SO_4^{2-}	1.322(1.139,1.534)	<0.001***	1.175(1.081,1.276)	<0.001***	1.260(1.114,1.426)	<0.001***	0.060	0.190	0.270

Table S8-3. Results of the stratified analyses.(Cross-sectional study)

Pollutants	drink	*0 vs 1*	smoke	*0 vs 1*

class	(IQR)	0	*P-value*	1	*P-value*	*Fisher’s permutation test*	0	*P-value*	1	*P-value*	*Fisher’s permutation test*
Respiratory class	$PM_{2.5}$	1.103(0.946,1.287)	0.235	1.149(0.985,1.342)	0.079	0.480	1.129(0.944,1.350)	0.216	1.119(0.976,1.283)	0.122	0.470
	PM_1	1.069(0.918,1.245)	0.422	1.149(0.985,1.341)	0.08	0.440	1.093(0.916,1.305)	0.382	1.105(0.964,1.267)	0.160	0.430
	PM_{10}	1.128(0.948,1.342)	0.179	1.194(0.999,1.426)	0.057	0.430	1.169(0.955,1.430)	0.154	1.157(0.989,1.354)	0.083	0.490
	NO_2	1.008(0.852,1.192)	0.971	1.057(0.896,1.247)	0.497	0.460	0.931(0.773,1.120)	0.381	1.093(0.939,1.273)	0.242	0.070
	SO_2	1.018(0.838,1.238)	0.887	1.218(0.998,1.487)	0.054	0.140	1.089(0.871,1.361)	0.495	1.119(0.936,1.337)	0.250	0.400
	O_3	1.010(0.857,1.190)	0.946	0.892(0.753,1.057)	0.19	0.090	1.034(0.848,1.262)	0.816	0.910(0.786,1.053)	0.209	0.170
	CO	1.187(0.996,1.414)	0.054	1.090(0.922,1.288)	0.339	0.170	1.083(0.894,1.311)	0.488	1.168(0.999,1.365)	0.061	0.230
	Cl^-	1.028(0.862,1.226)	0.813	1.082(0.914,1.281)	0.354	0.460	1.016(0.835,1.236)	0.905	1.063(0.910,1.242)	0.479	0.320
	NH_4^+	1.086(0.931,1.267)	0.294	1.212(1.032,1.422)	0.018*	0.290	1.063(0.888,1.271)	0.547	1.188(1.032,1.368)	0.019*	0.160
	NO_3^-	1.096(0.946,1.271)	0.231	1.138(0.979,1.322)	0.089	0.420	1.078(0.909,1.278)	0.434	1.136(0.993,1.299)	0.070	0.220
	SO_4^{2-}	1.269(1.066,1.510)	0.007**	1.207(1.014,1.436)	0.034*	0.260	1.137(0.928,1.392)	0.239	1.308(1.121,1.527)	0.001**	0.160
	$PM_{2.5}$	0.941(0.846,1.047)	0.247	1.006(0.897,1.128)	0.998	0.370	0.989(0.892,1.096)	0.772	0.950(0.844,1.069)	0.372	0.310
Stomach-arthritis class	PM_1	0.903(0.814,1.003)	0.054	0.969(0.866,1.084)	0.505	0.430	0.962(0.869,1.065)	0.418	0.903(0.804,1.013)	0.076	0.220
	PM_{10}	0.939(0.833,1.059)	0.316	0.991(0.870,1.129)	0.804	0.410	0.995(0.885,1.117)	0.919	0.925(0.807,1.060)	0.241	0.240
	NO_2	**0.867(0.775,0.969)**	**0.008****	**0.989(0.877,1.116)**	**0.774**	**0.040***	0.951(0.855,1.059)	0.268	0.889(0.785,1.008)	0.060	0.230
	SO_2	0.933(0.813,1.072)	0.28	0.958(0.827,1.110)	0.517	0.450	0.965(0.845,1.103)	0.531	0.914(0.784,1.066)	0.246	0.330
	O_3	0.883(0.790,0.987)	0.019*	0.938(0.832,1.057)	0.229	0.290	0.893(0.799,0.998)	0.029*	0.935(0.829,1.054)	0.221	0.210
	CO	1.009(0.901,1.129)	0.918	1.075(0.954,1.211)	0.25	0.250	1.070(0.961,1.191)	0.223	0.992(0.875,1.124)	0.878	0.210
	Cl^-	0.883(0.785,0.993)	0.038*	0.947(0.837,1.071)	0.329	0.350	**0.978(0.874,1.093)**	**0.659**	**0.831(0.729,0.947)**	**0.005****	**0.030***
	NH_4^+	0.924(0.830,1.030)	0.19	0.957(0.851,1.076)	0.422	0.470	0.953(0.857,1.060)	0.420	0.923(0.818,1.041)	0.190	0.380
	NO_3^-	0.981(0.883,1.089)	0.752	0.979(0.875,1.095)	0.625	0.350	0.998(0.902,1.104)	0.934	0.957(0.851,1.075)	0.456	0.330
	SO_4^{2-}	1.014(0.905,1.136)	0.714	1.113(0.978,1.267)	0.095	0.300	1.040(0.928,1.166)	0.376	1.073(0.945,1.219)	0.257	0.440
Vascular class	$PM_{2.5}$	**1.193(1.104,1.289)**	**<0.001*****	**1.393(1.273,1.524)**	**<0.001*****	**0.030***	1.275(1.181,1.377)	<0.001***	1.278(1.167,1.399)	<0.001***	0.450
	PM_1	**1.224(1.132,1.323)**	**<0.001*****	**1.401(1.281,1.533)**	**<0.001*****	**<0.001*****	1.324(1.225,1.431)	<0.001***	1.264(1.156,1.383)	<0.001***	0.260
	PM_{10}	**1.170(1.074,1.276)**	**<0.001*****	**1.346(1.219,1.485)**	**<0.001*****	**0.04***	1.232(1.132,1.340)	<0.001***	1.262(1.140,1.396)	<0.001***	0.400

NO_2	1.258(1.154,1.371)	<0.001***	1.334(1.211,1.469)	<0.001***	0.240	1.330(1.222,1.447)	<0.001***	1.248(1.132,1.376)	<0.001***	0.070
SO_2	1.216(1.103,1.340)	<0.001***	1.327(1.189,1.480)	<0.001***	0.180	1.293(1.175,1.422)	<0.001***	1.234(1.102,1.381)	<0.001***	0.230
O_3	1.104(1.014,1.202)	0.017*	1.149(1.044,1.265)	0.004**	0.260	1.138(1.045,1.240)	0.002**	1.107(1.007,1.218)	0.032*	0.280
CO	1.139(1.049,1.236)	0.002**	1.193(1.090,1.307)	<0.001***	0.260	1.166(1.076,1.263)	<0.001***	1.163(1.059,1.278)	0.001**	0.440
Cl^-	1.258(1.153,1.373)	<0.001***	1.357(1.232,1.494)	<0.001***	0.250	1.317(1.209,1.434)	<0.001***	1.286(1.164,1.421)	<0.001***	0.400
NH_4^+	**1.196(1.107,1.292)**	**<0.001*** **	**1.341(1.227,1.466)**	**<0.001*** **	**0.040***	1.275(1.181,1.378)	<0.001***	1.233(1.128,1.347)	<0.001***	0.230
NO_3^-	**1.242(1.151,1.340)**	**<0.001*** **	**1.380(1.265,1.506)**	**<0.001*** **	**0.040***	1.312(1.217,1.414)	<0.001***	1.284(1.176,1.403)	<0.001***	0.220
SO_4^{2-}	**1.149(1.060,1.246)**	**0.001***	**1.335(1.212,1.471)**	**<0.001*** **	**<0.001*** **	1.196(1.102,1.299)	<0.001***	1.263(1.148,1.388)	<0.001***	0.350

*P < 0.05; **P < 0.01; ***P < 0.001

Notes: Abbreviations:IQR, inter-quartile range.

Adjusted:age, sex, meteorological factors (temperature, relative humidity), marital status, education state, health status, patients' BMI, place of residence, social activity, public insurance, lifestyle habits (including alcohol consumption, smoking), cooking fuel, economic region category, depression, physical activity.

Impact estimates have been calculated for each quartile increment in the 3-year mean concentration of air contaminants.

Table S9-1. Results of the stratified analyses.(Longitudinal study)

pollutants	age				<60 *vs* >=60	sex				male *vs* *f*emale
	<60	*P-value*	>=60	*P-value*	*Fisher’s permutation test*	male	*P-value*	female	*P-value*	*Fisher’s permutation test*
$PM_{2.5}$	1.192(0.976,1.455)	0.101	1.213(0.982,1.497)	0.066	0.410	1.331(1.058,1.673)	0.018*	1.101(0.913,1.326)	0.253	0.420
PM_1	1.311(0.927,1.854)	0.148	1.425(0.983,2.066)	0.055	0.450	1.685(1.119,2.537)	0.016*	1.174(0.849,1.623)	0.274	0.210
PM_{10}	1.128(0.995,1.280)	0.072	1.113(0.981,1.263)	0.088	0.430	1.191(1.036,1.369)	0.017*	1.061(0.945,1.191)	0.256	0.380
NO_2	1.358(0.971,1.900)	0.084	1.465(1.056,2.032)	0.023*	0.340	1.825(1.260,2.645)	0.002**	1.236(0.903,1.693)	0.166	0.380
SO_2	2.727(1.731,4.296)	<0.001***	4.244(2.422,7.435)	<0.001***	0.410	4.318(2.396,7.782)	<0.001***	2.789(1.807,4.304)	<0.001***	0.390
O_3	0.787(0.625,0.991)	0.037*	0.783(0.630,0.973)	0.03*	0.460	0.779(0.593,1.024)	0.077	0.783(0.648,0.946)	0.016*	0.420
CO	1.260(1.079,1.471)	0.003**	1.277(1.101,1.481)	0.001**	0.490	1.306(1.117,1.526)	0.001**	1.240(1.073,1.435)	0.003**	0.470
Cl^-	1.274(0.846,1.917)	0.273	1.588(0.892,2.827)	0.107	0.490	1.789(1.045,3.063)	0.039*	1.113(0.734,1.687)	0.524	0.310
NH_4^+	1.125(0.969,1.306)	0.14	1.098(0.945,1.275)	0.198	0.490	1.158(0.985,1.361)	0.087	1.057(0.920,1.215)	0.35	0.450
NO_3^-	1.040(0.961,1.126)	0.389	1.027(0.954,1.105)	0.447	0.500	1.069(0.982,1.162)	0.143	1.006(0.938,1.079)	0.731	0.390
SO_4^{2-}	1.120(0.990,1.268)	0.124	1.105(0.984,1.242)	0.182	0.480	1.140(1.007,1.291)	0.014*	1.071(0.954,1.202)	0.252	0.450

Table S9-2. Results of the stratified analyses.(Longitudinal study)

pollutants	health						*0 vs 1*	*0 vs 2*	*1 vs 2*
	0	*P-value*	1	*P-value*	2	*P-value*	*Fisher’s permutation test*	*Fisher’s permutation test*	*Fisher’s permutation test*
$PM_{2.5}$	1.193(0.935,1.521)	0.12	1.140(0.921,1.411)	0.199	1.509(0.989,2.303)	0.057	0.390	0.440	0.240
PM_1	1.361(0.890,2.081)	0.202	1.246(0.858,1.811)	0.212	2.162(1.020,4.582)	0.045*	0.320	0.440	0.250
PM_{10}	1.127(0.967,1.314)	0.096	1.078(0.946,1.228)	0.218	1.251(0.980,1.597)	0.072	0.330	0.400	0.310

NO_2	1.466(0.981,2.191)	0.11	1.379(0.977,1.946)	0.078	2.031(0.999,4.128)	0.05	0.500	0.470	0.130
SO_2	3.032(1.814,5.068)	<0.001***	4.679(2.542,8.611)	<0.001***	3.009(0.925,9.787)	0.068	0.500	0.280	0.430
O_3	0.825(0.639,1.064)	0.149	0.762(0.608,0.956)	0.02*	1.142(0.640,2.040)	0.65	0.400	0.340	0.340
CO	1.328(1.095,1.609)	0.003**	1.252(1.067,1.469)	0.006**	1.375(1.013,1.865)	0.043*	0.430	0.460	0.340
Cl^-	1.477(0.859,2.537)	0.124	1.186(0.713,1.971)	0.478	2.390(0.919,6.216)	0.081	0.400	0.500	0.340
NH_4^+	1.099(0.927,1.304)	0.228	1.066(0.914,1.245)	0.372	1.366(0.967,1.930)	0.083	0.340	0.480	0.170
NO_3^-	1.032(0.945,1.127)	0.419	1.018(0.941,1.100)	0.616	1.167(0.975,1.397)	0.097	0.250	0.470	0.240
SO_4^{2-}	1.115(0.958,1.298)	0.162	1.058(0.941,1.190)	0.392	1.256(0.976,1.616)	0.068	0.420	0.420	0.130

Table S9-3. Results of the stratified analyses.(Longitudinal study)

pollutants	drink				*0 vs 1*	smoke				*0 vs 1*
	0	*P-value*	1	*P-value*	*Fisher's permutation test*	0	*P-value*	1	*P-value*	*Fisher's permutation test*
$PM_{2.5}$	1.192(0.976,1.455)	0.101	1.213(0.982,1.497)	0.066	0.380	1.331(1.058,1.673)	0.018*	1.101(0.913,1.326)	0.253	0.360
PM_1	1.311(0.927,1.854)	0.148	1.425(0.983,2.066)	0.055	0.210	1.685(1.119,2.537)	0.016*	1.174(0.849,1.623)	0.274	0.300
PM_{10}	1.128(0.995,1.280)	0.072	1.113(0.981,1.263)	0.088	0.190	1.191(1.036,1.369)	0.017*	1.061(0.945,1.191)	0.256	0.350
NO_2	1.358(0.971,1.900)	0.084	1.465(1.056,2.032)	0.023*	0.240	1.825(1.260,2.645)	0.002**	1.236(0.903,1.693)	0.166	0.200
SO_2	2.727(1.731,4.296)	<0.001***	4.244(2.422,7.435)	<0.001***	0.210	4.318(2.396,7.782)	<0.001***	2.789(1.807,4.304)	<0.001***	0.480
O_3	0.787(0.625,0.991)	0.037*	0.783(0.630,0.973)	0.03*	0.440	0.779(0.593,1.024)	0.077	0.783(0.648,0.946)	0.016*	0.380
CO	1.260(1.079,1.471)	0.003**	1.277(1.101,1.481)	0.001**	0.430	1.306(1.117,1.526)	0.001**	1.240(1.073,1.435)	0.003**	0.470
Cl^-	1.274(0.846,1.917)	0.273	1.588(0.892,2.827)	0.107	0.250	1.789(1.045,3.063)	0.039*	1.113(0.734,1.687)	0.524	0.260
NH_4^+	1.125(0.969,1.306)	0.14	1.098(0.945,1.275)	0.198	0.440	1.158(0.985,1.361)	0.087	1.057(0.920,1.215)	0.35	0.310

NO_3^-	1.040(0.961,1.126)	0.389	1.027(0.954,1.105)	0.447	0.410	1.069(0.982,1.162)	0.143	1.006(0.938,1.079)	0.731	0.250
SO_4^{2-}	1.120(0.990,1.268)	0.124	1.105(0.984,1.242)	0.182	0.460	1.140(1.007,1.291)	0.014*	1.071(0.954,1.202)	0.252	0.420

*P < 0.05; **P < 0.01; ***P < 0.001

Notes: Adjusted:age, sex, meteorological factors (temperature, relative humidity), marital status, education state, health status, patients' BMI, place of residence, social activity, public insurance, lifestyle habits (including alcohol consumption, smoking), cooking fuel, economic region category, depression, physical activity.

Air pollutants $PM_{2.5}$, PM_1, PM_{10}, NO_2, SO_2 (increasing by 10 μg/m3 each), CO (increasing by 0.1 mg/m3), and PMC (Cl^-, NH_4^+, NO_3^-, SO_4^{2-}) (increasing by 1 μg/m3 each) are used to calculate the impact estimate.

Impact estimates have been calculated for each quartile increment in the 3-year mean concentration of air contaminants.

Table S10. Sensitivity analyses of exposure to air pollutants and risk of MCC patterns(Cross-sectional study)

Class group	pollution (IQR)	model 1	*P-value*	model 2	*P-value*	model 3	*P-value*
respiratory class (n=384)	$PM_{2.5}$	1.144 (1.026 ,1.275)	0.015*	1.095 (0.957 ,1.253)	0.187	1.104 (0.980 ,1.243)	0.104
	PM_1	1.063 (0.957 ,1.181)	0.251	1.090 (0.950 ,1.250)	0.218	1.105 (0.981 ,1.246)	0.100
	PM_{10}	1.143 (1.013 ,1.290)	0.030*	1.131 (0.967 ,1.323)	0.123	1.147 (1.001 ,1.315)	0.049*
	O_3	0.952 (0.846 ,1.071)	0.414	0.890 (0.766 ,1.035)	0.131	0.962 (0.845 ,1.095)	0.559
	NO_2	1.037 (0.923 ,1.165)	0.538	0.967 (0.835 ,1.119)	0.649	1.032 (0.908 ,1.173)	0.627
	SO_2	1.146 (1.000 ,1.314)	0.051	1.049 (0.882 ,1.247)	0.591	1.102 (0.946 ,1.283)	0.213
	CO	1.119 (0.998 ,1.255)	0.054	1.121 (0.963 ,1.306)	0.141	1.145 (1.003 ,1.306)	0.045*
	Cl^-	1.040 (0.917 ,1.179)	0.543	0.995 (0.855 ,1.159)	0.953	1.051 (0.920 ,1.201)	0.462
	$NH4^+$	1.156 (1.036 ,1.290)	0.010*	1.143 (0.994 ,1.315)	0.060	1.121 (0.994 ,1.265)	0.063
	$NO3^-$	1.135 (1.019 ,1.264)	0.022*	1.119 (0.980 ,1.279)	0.097	1.109 (0.987 ,1.246)	0.083
	$SO4^{2-}$	1.209 (1.076 ,1.359)	0.001**	1.274 (1.090 ,1.489)	0.002**	1.231 (1.076 ,1.409)	0.002**
stomach-arthritis class (n=934)	$PM_{2.5}$	0.944 (0.874 ,1.019)	0.140	0.920 (0.832 ,1.018)	0.105	0.958 (0.879 ,1.044)	0.332
	PM_1	0.912 (0.847 ,0.982)	0.014*	0.895 (0.810 ,0.989)	0.029*	0.933 (0.858 ,1.015)	0.108
	PM_{10}	0.944 (0.865 ,1.030)	0.193	0.945 (0.842 ,1.061)	0.339	0.968 (0.877 ,1.068)	0.514
	O_3	0.902 (0.831 ,0.980)	0.014*	0.879 (0.792 ,0.976)	0.016*	0.911 (0.832 ,0.997)	0.043*
	NO_2	0.887 (0.818 ,0.962)	0.004**	0.867 (0.780 ,0.963)	0.008**	0.926 (0.846 ,1.013)	0.094
	SO_2	0.961 (0.871 ,1.059)	0.423	0.921 (0.808 ,1.050)	0.218	0.940 (0.841 ,1.050)	0.273
	CO	1.023 (0.946 ,1.106)	0.569	1.018 (0.917 ,1.130)	0.745	1.045 (0.953 ,1.145)	0.349
	Cl^-	0.892 (0.818 ,0.973)	0.010**	0.898 (0.805 ,1.003)	0.056	0.901 (0.820 ,0.990)	0.030*
	$NH4^+$	0.939 (0.868 ,1.016)	0.117	0.923 (0.832 ,1.024)	0.132	0.935 (0.857 ,1.021)	0.134
	$NO3^-$	0.964 (0.893 ,1.042)	0.359	0.960 (0.869 ,1.062)	0.432	0.982 (0.901 ,1.069)	0.668
	$SO4^{2-}$	1.065 (0.982 ,1.155)	0.131	1.041 (0.933 ,1.162)	0.471	1.045 (0.951 ,1.148)	0.356
relatively healthy class (n=1701)	$PM_{2.5}$	1.282 (1.210 ,1.359)	<0.001***	1.250 (1.168 ,1.337)	<0.001***	1.250 (1.172 ,1.335)	<0.001***
	PM_1	1.277 (1.206 ,1.353)	<0.001***	1.283 (1.198 ,1.375)	<0.001***	1.274 (1.193 ,1.361)	<0.001***
	PM_{10}	1.233 (1.156 ,1.314)	<0.001***	1.224 (1.135 ,1.320)	<0.001***	1.220 (1.135 ,1.312)	<0.001***
	O_3	1.107 (1.038 ,1.180)	0.002**	1.137 (1.055 ,1.225)	0.001**	1.155 (1.076 ,1.241)	<0.001***
	NO_2	1.269 (1.192 ,1.352)	<0.001***	1.270 (1.178 ,1.370)	<0.001***	1.296 (1.206 ,1.393)	<0.001***
	SO_2	1.263 (1.175 ,1.356)	<0.001***	1.242 (1.141 ,1.351)	<0.001***	1.242 (1.145 ,1.347)	<0.001***
	CO	1.147 (1.082 ,1.216)	<0.001***	1.150 (1.071 ,1.235)	<0.001***	1.149 (1.072 ,1.231)	<0.001***
	Cl^-	1.287 (1.205 ,1.375)	<0.001***	1.273 (1.180 ,1.373)	<0.001***	1.285 (1.194 ,1.382)	<0.001***
	$NH4^+$	1.250 (1.181 ,1.324)	<0.001***	1.242 (1.160 ,1.329)	<0.001***	1.241 (1.163 ,1.325)	<0.001***
	$NO3^-$	1.278 (1.207 ,1.353)	<0.001***	1.293 (1.210 ,1.383)	<0.001***	1.292 (1.212 ,1.377)	<0.001***
	$SO4^{2-}$	1.206 (1.137 ,1.280)	<0.001***	1.210 (1.126 ,1.300)	<0.001***	1.207 (1.126 ,1.293)	<0.001***

*Notes:**P < 0.05; **P < 0.01; ***P < 0.001

Abbreviations:*IQR,* inter-quartile range.

In Group 2 and Group 3 ,impact estimates have been calculated for each quartile increment in the 3-year mean concentration of air contaminants.

1) Group 1: The exposure concentrations of air pollutants are two-year averaged.

2) Group 2: Excluded participants in very poor health.

3) Group 3: Excluded missing values for age,bmi, place of residence,public insurance,social activity, and physical activity level at baseline.

Table S11. Sensitivity analyses of exposure to air pollutants and risk of MCC (Longitudinal study)

pollution（IQR）	Group 1	*p-value*	Group 2	*p-value*	Group 3	*p-value*
$PM_{2.5}$	1.176(1.024,1.351)	0.002**	1.162(1.001,1.350)	0.045*	1.225(1.054,1.424)	<0.001***
PM_1	1.312(1.029,1.673)	0.002**	1.293(0.996,1.678)	0.076	1.433(1.100,1.866)	<0.001***
PM_{10}	1.133(1.040,1.234)	<0.001***	1.100(1.003,1.207)	0.032*	1.130(1.031,1.238)	<0.001***
NO_2	1.570(1.259,1.957)	<0.001***	1.405(1.096,1.802)	<0.001***	1.432(1.126,1.821)	<0.001***
SO_2	4.430(2.932,6.693)	<0.001***	3.233(2.275,4.594)	<0.001***	3.131(2.211,4.435)	<0.001***
O_3	0.821(0.710,0.949)	0.011*	0.801(0.681,0.943)	<0.001***	0.821(0.696,0.969)	<0.001***
CO	1.316(1.175,1.473)	<0.001***	1.276(1.138,1.431)	<0.001***	1.275(1.142,1.423)	<0.001***
Cl^-	1.275(0.931,1.745)	0.084	1.320(0.934,1.865)	0.056	1.443(1.023,2.035)	<0.001***
NH_4^+	1.090(0.986,1.205)	0.104	1.082(0.973,1.205)	0.108	1.130(1.011,1.263)	0.002**
NO_3^-	1.026(0.975,1.080)	0.349	1.024(0.970,1.081)	0.372	1.043(0.986,1.103)	0.122
SO_4^{2-}	1.105(1.017,1.200)	0.03*	1.084(1.007,1.184)	0.033**	1.164(1.022,1.218)	0.006**

*Notes:**P < 0.05; **P < 0.01; ***P < 0.001

In Group 2 and Group 3 ,impact estimates have been calculated for each quartile increment in the 3-year mean concentration of air contaminants.

1)Group 1: The exposure concentrations of air pollutants are two-year averaged.

2)Group 2: Excluded participants in very poor health.

3)Group 3: Excluded missing values for age,bmi, place of residence,public insurance,social activity, and physical activity level at baseline

Air pollutants $PM_{2.5}$, PM_1, PM_{10}, NO_2, SO_2 (increasing by 10 μg/m3 each), CO (increasing by 0.1 mg/m3), and PMC (Cl^-, NH_4^+, NO_3^-, SO_4^{2-}) (increasing by 1 μg/m3 each) are used to calculate the impact estimate.
